# Analysis of risk factors, clinical data, treatment outcomes for cats with feline infectious peritonitis using GS-441524 (2020–2024)

**DOI:** 10.1038/s41598-025-30674-2

**Published:** 2025-12-08

**Authors:** Maneli Ansari Mood

**Affiliations:** Riko Animal Polyclinic, Tehran, Islamic Republic of Iran

**Keywords:** GS-441524 efficacy, Antiviral therapy, Nucleoside analogue, Survival analysis, Feline coronavirus, Ocular FIP, Neurological FIP, Diseases, Experimental models of disease, Clinical trial design

## Abstract

Feline infectious peritonitis (FIP) is a lethal, immune-mediated disease caused by feline coronavirus (FCoV). FIP was considered untreatable; however, GS-441524, a nucleoside analog, has become a hopeful antiviral treatment. Despite its effectiveness, survival outcomes depend on several prognostic factors, especially the type of disease and clinical presentation. This research aimed to assess the effectiveness of GS-441524 in a large population of cats with FIP in Iran, examine survival rates, and identify crucial prognostic factors affecting treatment outcomes. Additionally, it evaluates alterations in clinical, laboratory, and imaging outcomes during treatment, proposing a secure treatment protocol for veterinarians utilizing GS-441524 for FIP. This retrospective study analyzed 629 cats diagnosed with or highly suspected of having FIP in Iran between December 2020 and March 2024. Diagnosis was based on clinical signs, laboratory findings, ultrasonographic features, and therapeutic responses. Cats received GS-441524 via subcutaneous injection and/or oral administration for a minimum of 12 weeks. Dosages were adjusted according to the FIP form, and clinical, laboratory, and imaging data were collected before, during, and at the end of treatment with GS-441524. Dosages were further adjusted based on FIP form, weight gain, clinical improvement, and laboratory or imaging results. Statistical analyses comprised ANOVA, t-tests, chi-square tests, and non-parametric techniques to pinpoint important prognostic indicators and treatment effects. The survival rate reached 94.12%, with a relapse rate of 0.63%. Most reported type was Effusive forms accounted for 54.84% of the cats. Key prognostic factors associated with reduced survival included being male, over 6 years old, having neurological or mixed forms of FIP, and exhibiting fever, icterus, anemia, and thrombocytopenia (*p* < 0.05). Significant improvements were observed in parameters such as the A: G ratio, albumin, globulin, bilirubin levels, and changing in imaging findings. The study also assessed dosage modifications during treatment. The average starting dose for effusive forms was 6.9 mg/kg, later increasing to 10.11 mg/kg, while neurological and mixed forms initially required 9.65 mg/kg, which was then raised to 12.7 mg/kg. Treatment duration extended beyond 84 days for 17.32% of cats (109 cats) due to showing abnormalities. Imaging studies confirmed a gradual resolution of abdominal and pleural effusions, reduced lymph node size (both abdominal and mediastinal), decreased kidney size in cats of renomegaly, and regression of gallbladder edema. This research features the largest group of FIP-treated cats in Iran and ranks among the largest worldwide, showcasing impressive survival rates with GS-441524 treatment. Positive results rely on timely intervention, suitable dosage modifications, and extended treatment periods, particularly for cats showing neurological and ocular signs. Major risk factors affecting lower survival rates were fever, icterus, anemia, and low platelet count. These findings provide vital clinical insights for veterinarians, underscoring the need for customized treatment approaches and continued research to enhance FIP outcomes therapy and long-term care.

## Introduction

Feline Infectious Peritonitis (FIP) is a fatal, immune-mediated disease caused by certain strains of the feline coronavirus (FCoV). While most cats infected with FCoV remain asymptomatic or exhibit mild gastrointestinal symptoms, a small percentage develop FIP due to a virus mutation^1,2^. This mutation causes changes in tissue or cell tropism in the virus, leading to alterations in virulence and the characteristics of the disease. In FIP, the virus acquires tropism for monocytes and macrophages, resulting in fatal diseases^3^. The disease exhibits a spectrum of clinical presentations, including an effusive (“wet”) form and a non-effusive (“dry”) form, both of which may be complicated by neurological or ocular involvement. Depending on the form and the affected organs, FIP can manifest clinically with a wide range of symptoms. As a result, diagnosing FIP is challenging due to its complex pathogenesis, variable clinical presentations, and the lack of a single definitive diagnostic method test^2,3^. For many years, finding an effective treatment was challenging. All cats with FIP either die or have to be euthanized because of no effective treatment, and the average survival time after the presentation of symptoms could be from weeks to months^4^. However, recent advancements have identified GS-441524, a nucleoside analog, as a promising antiviral agent for treating FIP. GS-441524 is an antiviral nucleotide analog developed by Gilead Sciences and closely related to remdesivir (GS-5734)^5,6^. While remdesivir has received widespread use in humans, GS-441524 has not been approved for human or veterinary use in most regions. These analogs serve as alternative substrates and RNA-chain terminators of viral RNA-dependent RNA polymerase, reducing viral load and mitigating disease progression. Murphy et al.^7^ determined that GS-441524 was non-toxic to feline cells at concentrations as high as 100 μM and effectively inhibited FIPV replication in cultured CRFK cells and naturally infected feline peritoneal macrophages at concentrations as low as 1 μM. Several other studies in recent years showed the efficacy of GS-441524 in treating FIP in cats^4,5,7–9^. It is a predominant metabolite of Remdesivir and, in fact, the parent nucleoside of Remdesivir, GS-441524, has advantages over Remdesivir itself for treating COVID-19^10^. Pederson et al. 2019 illustrated GS-441524 as a safe and effective treatment for FIP. The optimum dosage was 4.0 mg/kg SC q24h for at least 12 weeks, and 18 of these 26 cats remained healthy at the time of publication^4^0.18 cats with diagnosed FIP were effectively treated for 12 weeks with the antiviral drug Xraphconn (Mutian), which contains GS-441524, in the first controlled prospective oral treatment investigation^9^. Cosaro et al.^11^ demonstrated the efficacy of orally administered GS-441514 (12.5–15 mg/kg) for treatment. Taylor et al.^12^ reported that legally sourced remdesivir and GS-441524 products, either alone or used sequentially, were very effective in treating FIP, and of 307 cats, 84.4% were alive. Dickinson et al.^13^ reported four cases of naturally occurring FIP with central nervous system (CNS) involvement that were treated with GS-441524 (5–10 mg/kg) for at least 12 weeks. Additionally, a study showed orally administered GS-441524, given as a short treatment, was highly effective in curing FIP without causing serious problems^14^. The main problem is that GS-441524 has not yet been approved in most countries (except the UK, Australia, and, more recently, Canada and the United States), creating a black market^8,14^. As a result, it is not easily accessible, and the cost of treatment has increased.

This research aimed to demonstrate the effectiveness of GS-441524 through oral or injection therapy for a large population of cats with highly suspected or confirmed FIP and a long post-recovery follow-up period, moreover, intended to present a standardized treatment strategy and identify the factors that may contribute to successful treatment outcomes for veterinarians, determine the survival rate, and observe the clinical, laboratory, and imaging findings in FIP and how they change during treatment. This study is also the first report on FIP treatment with GS-441524 in Iran and one of the most extensive global studies involving cats diagnosed with FIP.

## Material and methods

### Study design: the study design was a retrospective analysis of medical records

Case selection: Data were collected from December 2020 to March 2024. A total of 629 cats were diagnosed with or highly suspected of FIP in Iran, most of which were from Tehran, the capital, and included referral cats from other cities. This group comprised 412 males and 217 females, aged between 2.5 months and 10 years. The initial diagnosis of FIP was based on clinical signs, disease signalment, physical findings, routine laboratory test results, examination of abdominal or thoracic effusions, and sonographic findings^2,15,16^. In effusive (wet) FIP, diagnosis is facilitated by fluid aspiration and analysis, which provide characteristic cytologic and biochemical findings. The main issue is with the dry form. Clinical signs such as anorexia, lethargy, weight loss, pyrexia unresponsive to antibiotic treatment, ocular and neurological signs (as mentioned in Table 2) are non-specific but warrant further investigation. In such cases, supportive diagnostic indicators include anemia, hyperglobulinemia, an A:G ratio less than 0.5, abdominal lymphadenopathy, and renomegaly, which help detect FIP in dry forms^2,15^.

A more definitive diagnosis, such as immunohistochemistry, is not available in Iran and is invasive. PCR testing and rapid FIP tests (antibody or antigen detecting) were not used as the primary basis for diagnosis, as these tests detect FCoV. Nevertheless, some tests were conducted on certain cats; however, positive results for FIP were interpreted in conjunction with history, clinical, laboratory, and imaging findings.

Information regarding each cat includes the type of FIP, age, gender, breed, sterilization status, weight fluctuations, whether the cat was indoor or outdoor, and whether it lives in a single or multi-cat household. Additional recorded information includes body temperature, appetite level, lethargy, icterus (jaundice), abdominal swelling, rapid or difficulty breathing, diarrhea, grinding of teeth, and neurological and eye examinations. Additionally, any stress factors and a history of illnesses from the past month for each cat were recorded and documented. Concomitant diseases such as feline immunodeficiency virus (FIV), feline leukemia virus (FeLV), Mycoplasma haemofelis, cardiopathy, renal diseases, diabetes, toxoplasmosis, panleukopenia (FPV), pancreatitis, gingivitis, asthma, and upper respiratory diseases were also recorded. In addition, a complete blood count (CBC) was performed, along with biochemical measurements of total protein (TP), albumin (Alb), globulin (Glu), albumin-to-globulin A/G ratio, total bilirubin (TB), blood urea nitrogen (BUN), serum creatinine (Cr), alanine transaminase (ALT), aspartate transaminase (AST), and alkaline phosphatase (ALP).

Abdominal ultrasonography (US) was performed on each cat, documenting changes in internal organs and mesentery condition, including fluid buildup and lymph node (LN) alterations. Thoracic radiographs were taken to evaluate for pleural effusion, enlarged sternal lymph nodes, and changes in lung pattern for those showing respiratory signs such as dyspnea, tachypnea, panting, or any thoracic auscultation abnormalities. Advanced imaging, Magnetic resonance imaging (MRI), was performed on three cats with neurological signs to confirm FIP.

Cat data were used to categorize the types of FIP as follows:

1. Effusion—abdominal: fluid in the peritoneal and/or retroperitoneal space
2. Effusion—thoracic: fluid in the pleural and/or pericardial space
3. Effusion—abdominal and thoracic: 1 and 2 combined
4. Noneffusive: without effusion, displaying a granulomatous reaction in the abdomen
5. Ocular: ocular signs are prominent
6. Neurological: neurological signs are prominent
7. Neuro-ocular: exhibiting both neurological and ocular signs
8. Mixed form: effusion with neurological and/or ocular signs

### Treatment protocol

*Drug preparation*: Since GS-441524 was available only through the black market in Iran, different brands supplied it in vials with 15%, 20%, and 30% dilutions, as well as in tablet or capsule form, 20–50 mg.

Table 1 summarizes the initial and final dosages for each form. Dosages were chosen based on published studies with GS-441524, insights from FIP Warriors Facebook group veterinarians, and the author’s clinical observations^2,4,9,17^. During the final 21 days of treatment, the dosage was raised by 2–3 mg/kg; this adjustment occurred whenever treatment had to be prolonged due to disease relapse or worsening symptoms. GS-441524 was administered subcutaneously every 24 h, as scheduled. In some cats, the oral form was used in the continuation phase due to the high volume required for injection, injection-associated stress, skin ulceration at injection sites, or the owner’s preference for oral administration. Injection sites were rotated across six locations on the dorsum, ranging from 2 cm caudal to the scapulae to the mid-lumbar region on both sides of the vertebral column. This rotation aimed to minimize local pain and tissue irritation. Tablets were administered once daily at the same time on an empty stomach. Food was provided 1 h before and after administration to increase absorption and reduce gastrointestinal upset.

**Table 1:** Starting and ending dosage in different types of FIP.

FIP form	Starting dose (mg/kg)	Ending dose (mg/kg)
Effusion- abdominal	6–8	8–11
Effusion- thoracic	8	10–11
Effusion- abdominal/thoracic	8	10–11
None- effusive	9–10	11–13
Ocular	9–10	11–13
Neurological	10–12	12–17
Mixed	10–12	12–17

According to studies by Pedersen NC et al.^4,18^ and Taylor et al.^12^, the minimum treatment period in this protocol was set at 12 weeks^19^.

To determine the appropriate endpoint for GS-441524 treatment, four clinical categories were routinely assessed for each cat in this study, as detailed below:

- *Weight*: Gaining body weight (BW) during treatment is crucial, especially for kittens^18^. A key indicator of successful treatment is reaching a body condition score (BCS) of 4/10 or higher^20^. With effective treatment, kittens will gain weight and grow. In adult patients, we can compare body weight to when the cat was clinically normal. We can also compare the kittens to their healthy siblings regarding growth and weight gain for some cats.
- *Clinical signs*: Include improved fever, lethargy, weakness, icterus, anorexia, and depression. Although some patients show improved entirely symptoms in the ocular and neurological forms, in some of them, a full recovery is unlikely due to the progression of FIP and the late initiation of treatment. In these cats, while treating with an increasing dose of GS-441524 and providing supportive care to manage the persistent symptoms (uveitis, elevated intraocular pressure[IOP], seizures, urinary incontinence, etc.), we will extend the treatment duration and adjust the dosage to relieve these symptoms. However, if these symptoms persist and other indicators, such as weight, appetite, body temperature, and laboratory or imaging results, are normal, we might stop the treatment.
- *Laboratory findings*: Improvement in anemia, serum A: G ratio > 0.5, total bilirubin < 0.5 mg/dL, total protein < 8.2 g/dL, globulin ≤ 4.4 g/dL.
- *Imaging findings*: Resolution of fluid accumulation in cavities (pleural, abdominal, retroperitoneal), decreased lymph node size (abdominal, mediastinal), decreased kidney size in renomegaly form, and regression of gallbladder edema.

Based on the points mentioned above, the treatment for the cats was extended for one or more weeks.

### Monitoring the treatment

In the initial treatment phase, cats were removed from all non-essential medications, such as pentoxifylline and corticosteroids, previously used for their anti-inflammatory or analgesic effects purposes^4^. All treatments except GS-441524 were prescribed solely based on necessity, including therapy for Mycoplasma hemophilus, dehydration, severe anemia, FPV, and others. In this protocol, routine adjunctive therapy comprised vitamin B12, hepatic supplements like silymarin, and occasionally, omega-3. Supportive care was tailored to the clinical context and included antibiotics for infections such as Mycoplasma and Toxoplasma, post-surgical prophylaxis, fluid therapy, and corticosteroid administration. Gabapentin (5–10 mg/kg) was administered either for patient sedation prior to injection or as analgesia in required cats.

After initiating treatment, the cats were monitored daily for temperature, hydration, appetite, activity, urination, and defecation until they returned to normal conditions. Blood samples were also collected 3 to 7 days later from those receiving additional treatment or who were in critical condition. When a significant positive response to treatment was observed, follow-up evaluations were conducted on days 28, 56, and 83. Physical examinations, clinical signs, and previous laboratory and imaging diagnostic tests were reassessed. All changes were documented, as these findings were essential for managing treatment, response to treatment, and making dosage adjustments. In this protocol, the cats were evaluated on the 83rd day rather than the 84th day before concluding the treatment, as I reviewed the details to determine whether to stop or continue the treatment. Obviously, not all tests were performed accurately on schedule, as mentioned, but this survey did its best to adhere to the timeline. In addition to the examinations conducted at 4-week intervals, owners were required to monitor their cats and remain in regular contact with the veterinarian. Some intermediate physical examinations, laboratory tests, and imaging procedures were not performed due to the owner’s negligence, financial limitations, or lack of access to paraclinical service equipment. Despite these challenges, the author did not discontinue the treatment without completing the final laboratory and clinical examinations. Weekly body weight measurements were recorded to calculate the accurate dosage of GS-441524. The follow-up period (in days) after treatment ended was documented for each cat. The first 90 days following the final treatment point were designated as the observation period, which was critical for minimizing stressors that could increase the risk of FIP relapse in cats. Vaccination was not administered during treatment or for three months afterward, and it was recommended to wait until six months after the conclusion of treatment. However, antiparasitic therapy was not a concern and was administered as needed. Castration and ovariohysterectomy were performed in the indicated cats during the last 20 days of treatment. Continuing the treatment for at least 14 days following surgery was considered safer.

All data were initially logged in Microsoft Excel and then exported to SPSS for analysis (Version 26.0, IBM Corp., Armonk, NY, USA). Continuous variables, which included laboratory parameters and treatment dosages at various time points (Days 0, 28, 56, and 83), were assessed using one-way ANOVA and paired t-tests. To compare dosage levels and treatment responses between effusive and non-effusive forms of FIP, along with specific clinical subtypes like neurological and ocular manifestations, independent two-sample t-tests were utilized. Chi-square tests were employed to analyze categorical variables for associations between clinical findings, imaging results, and survival rates. For data that did not follow a normal distribution, the Kruskal–Wallis test was performed. A *p*-value of < 0.05 was considered statistically significant wa Independent two-sample t-tests were used to compare dosage levels and treatment responses between effusive and non-effusive FIP forms and among specific clinical subtypes such as neurological and ocular presentations. Categorical variables were evaluated using chi-square tests to assess associations between clinical findings, imaging results, and survival outcomes. For non-parametric data, the Kruskal-Walli’s test was applied. A *p*-value of < 0.05 was considered statistically significant in all analyses.

## Results

### Demographics

**Fig. 1:**
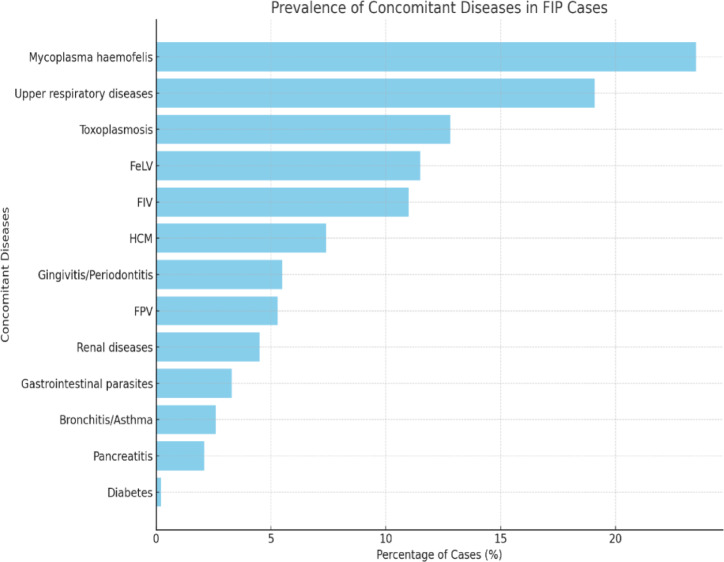
Prevalence of concomitant diseases observed in FIP cats.

Most cats, 88.7% (559 cats), originated from Tehran, the capital of Iran. Meanwhile, others came from various cities across the country, accounting for 11.13% (70 cats). Of the 629 cats, 412 (65.5%) were male, with 172 (27.34% of the total) neutered and 240 (38.16%) remaining intact. Conversely, 217 (34.5%) were female, including 73 spayed females (11.61% of the total) and 144 intact females (22.89% of the total). The average age at diagnosis was 1.1 years (approximately 13.2 months), with a standard deviation of 13.46 months. Ages ranged from 2.5 months to 10 years. Of the total, 76.31% (480 cats) were under 12 months old, 22.73% (143 cats) were between 12 months and 6 years, and 0.95% (6 cats) were older than 6 years. Data on breed was available for all 629 cats. Domestic Shorthair (DSH) cats made up 62.16% (391 cats), followed by Scottish Fold (117 cats, 18.6%), Persian (55 cats, 8.74%), British Shorthair (38 cats, 6.04%), Domestic Longhair (DLH) (9 cats, 1.43%), Himalayan (7 cats, 1.11%), mixed breed (5 cats, 0.8%), Siamese (5 cats, 0.8%), and Exotic Shorthair (2 cats, 0.32%). Regarding lifestyle, 60.57% (381 cats) lived indoors, 14.63% (92 cats) were outdoor cats, and 24.8% (156 cats) had access to both environments. Regarding household composition, 34.82% (219 cats) were the only cat in their homes, while 65.18% (410 cats) lived in multi-cat households. Outdoor cats were also categorized as part of multi-cat households. Data on potential stressors were collected from 557 cats in the month leading up to symptom onset. Of these, 45.1% (251 cats) had faced one or more stress events, including surgery, bathing, grooming, relocation, the introduction of a new cat, or vaccination. Regarding illness, among the 533 cats examined with available information, 8% (43 cats) had experienced FPV, 14.6% (78 cats) had URD, and 17.2% (92 cats) had diarrhea in the month prior to the onset of FIP symptoms. The other factor recorded was the presence of concomitant diseases with FIP. The disease most frequently reported was Mycoplasma Haemofelis, affecting 23.5% (148 cats), followed by upper respiratory diseases at 19.1% (120 cats). Other illnesses reported, in order of prevalence, included Toxoplasmosis at 12.8% (81 cats), FeLV at 11.5% (72 cats), FIV at 11% (69 cats), HCM at 7.4% (47 cats), gingivitis or periodontitis at 5.5% (35 cats), FPV at 5.3% (33 cats), renal diseases at 4.5% (28 cats), gastrointestinal parasites at 3.3% (21 cats), bronchitis or asthma at 2.6% (16 cats), pancreatitis at 2.1% (13 cats), and diabetes at 0.2% (1 cat). Only 158 FIP cats (25.11%) did not have any additional diseases during GS-441524 treatment. (Fig. 1).

### Disease characteristics

Out of 629 cats in this study, 201 cats (32%) had abdominal effusion, 37 cats (5.9%) had thoracic effusion, 38 cats (6%) had both abdominal and thoracic effusion, 94 cats (14.9%) had no effusion, 46 cats (7.3%) had ocular form, 30 cats (4.8%) had neurological form, 114 cats (18.1%) had neuro-ocular form, and 69 cats (11%) were mixed. (See Pie Chart 1).
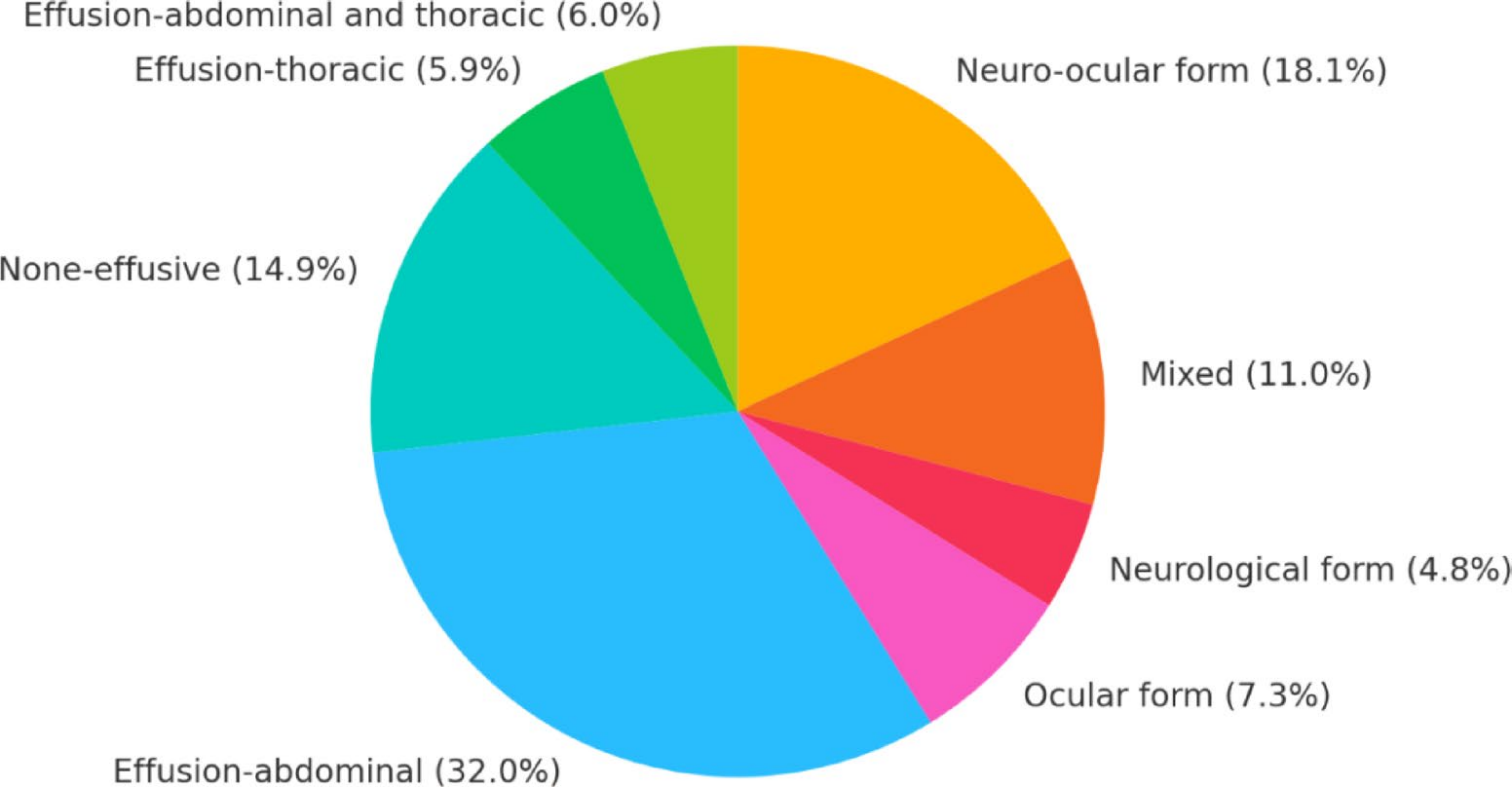


Lethargy was noted in 95.2% (599/629), reduced or absent appetite in 91.1% (573/629), weight loss in 89.17% (535/600), and fever (≥ 39.4 °C) in 80.29% (505/629). Additional clinical signs, physical examinations, and their occurrence rates are detailed in Table 2. According to the available data, the median duration of clinical signs observed before diagnosis was 14 days, ranging from 5 to 190 days. Fever remission (97.2%) and increased appetite and energy levels (74.52%) during the first week were the main markers of treatment success.

**Table 2:** Frequency of clinical signs and physical examination findings in cats diagnosed with FIP.

Clinical signs	Number/total	Percentage of cats (%)
Lethargy	599/629	95.2
Appetite loss	573/629	91.1
Weight loss	535/600	89.1
Fever	505/629	84.87
Icterus	253/629	40.22
Neurologic symptoms^a^	188/629	29.88
Ophthalmic symptoms^b^	227/629	36.1
Tachypnea/Dyspnea	75/629	11.92
Gastrointestinal symptoms^c^	33/629	5.2
Gnash^d^	11/629	1.7
Abdominal distention	232/629	36.88

^1^Seizures, posterior ataxia, paralysis, paresis, vestibular syndrome, incoordination, fecal and/or urinary incontinence, hyperesthesia, and nystagmus^2,18^.

^b^Uveitis, iritis, cloudiness, keratic precipitates, flocculent debris in the anterior chamber, anisocoria, glaucoma, panophthalmitis, chorioretinitis, hyphema, and hypopyon^18,21^.

^c^Diarrhea, vomiting.

^d^Move the teeth against each other.

The most frequently observed laboratory abnormalities in this study were a decreased A:G ratio (≤ 0.5) in 89.47% (561/627) of cats, elevated globulin levels in 86.92% (545/627), reduced albumin levels in 73.68% (462/627), anemia in 57.9% (363/627), lymphopenia in 50.88% (319/627), and thrombocytopenia in 43.22% (271/627). Additionally, elevated total protein (TP) levels were noted in 52.15% (327/627), while increased total bilirubin (bilirubinemia) was reported in 42.86% (261/609). Further details on additional laboratory findings are summarized in the accompanying Table 3.

**Table 3:** Laboratory abnormalities observed on Day 1 of treatment with GS-441524 in cats with FIP.

	Count; percentage	Normal range
Anemia (PCV < 24%)	363/627; 57.9%	24–45%
Leukocytosis (Leukocyte > 19,500/micL)	206/627; 32.85%	5500–19,500/micL
Leukocytopenia (Leukocyte < 5500/micL)	108/627; 17.22%	5500–19,500/micL
Lymphopenia (Lymphocyte < 1500/uL)	319/627; 50.88%	1500–7000/uL
Thrombocytopenia (Platelets < 175,000/micL)	271/627; 43.22%	175–600 × 10*3/micL
Increased Total Protein (TP ≥ 8.2 g/dL)	327/627; 52.15%	5.8–8.2 g/dL
Increased Globulin (G > 4.4 g/dL)	545/627; 86.92%	2.4–4.4 g/dL
Decreased Albumin (Alb < 2.4 g/dL)	462/627; 73.68%	2.4–3.9 g/dL
A/G ratio ≤ 0.5	561/627; 87.5%	0.6–1.5
Increased Total Bilirubin (> 0.5mg/dL)	261/609; 42.86%	0.15–0.5 mg/dL
Increased AST (> 48 U/L)	310/603; 51.4%	17–48 U/L
Increased ALT (> 97 U/L)	140/603; 23.21%	25–97 U/L
Increased ALP (> 93 U/L)	390/603; 64.67%	25–93 U/L
Increased BUN (> 36 mg/dL)	45/603; 7.46%	14–36 mg/dL
Increased Cr (> 1.8 mg/dL)	36/603; 5.97%	0.8–1.8 mg/dL

**Fig. 2:**
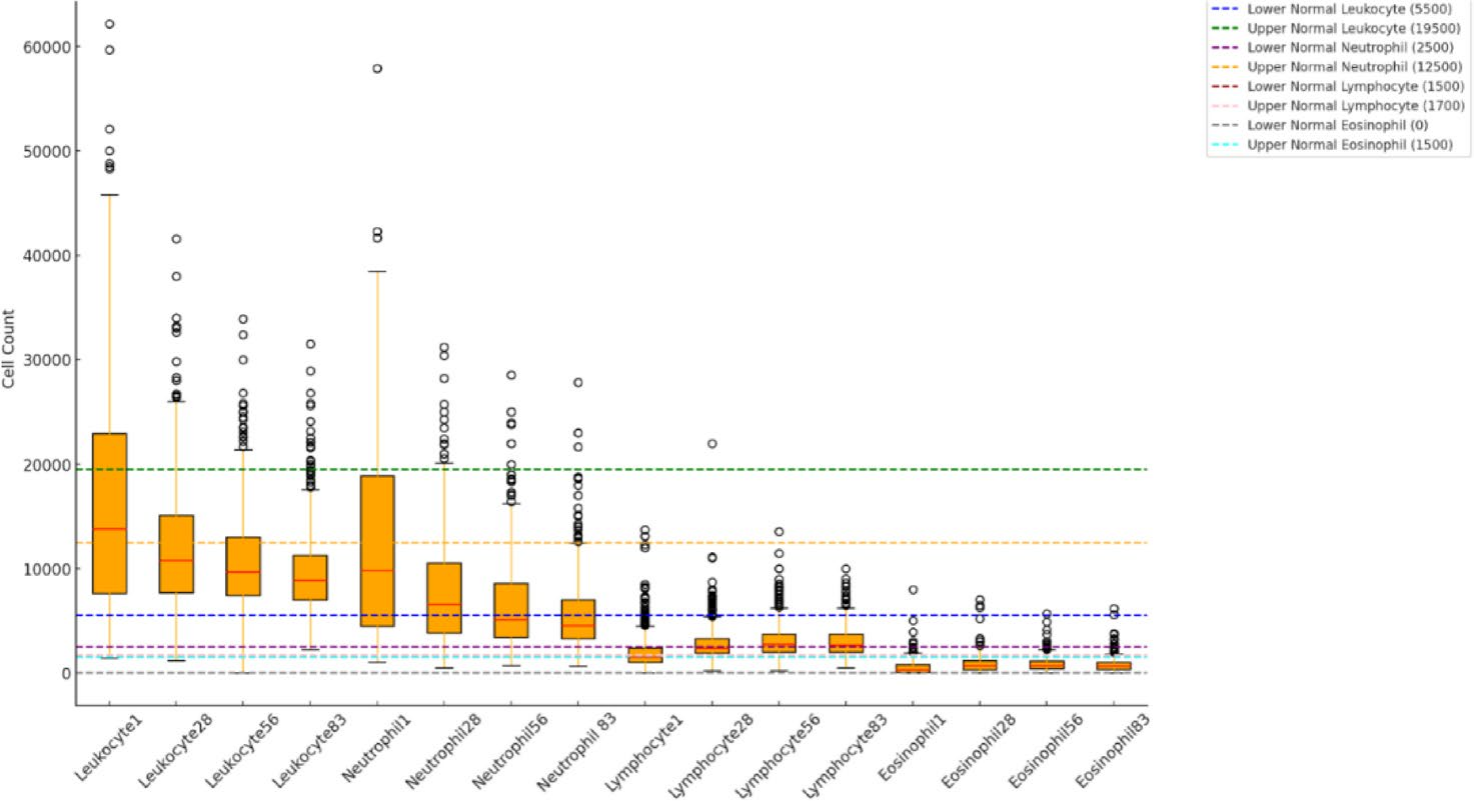
Leukocyte, neutrophil, lymphocyte, and eosinophil count across treatment time points.

**Fig. 3:**
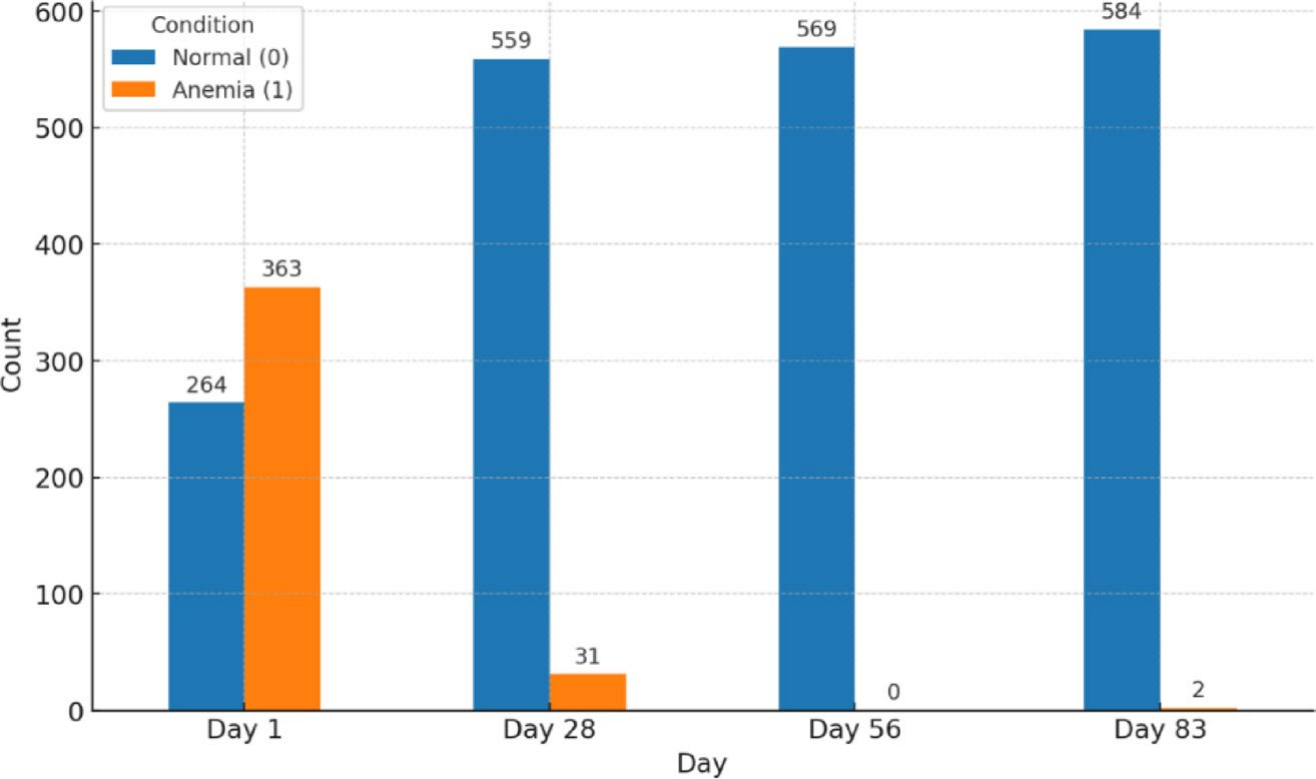
Anemia counts across treatment time points; Anemia (PCV < 24%).

**Fig. 4:**
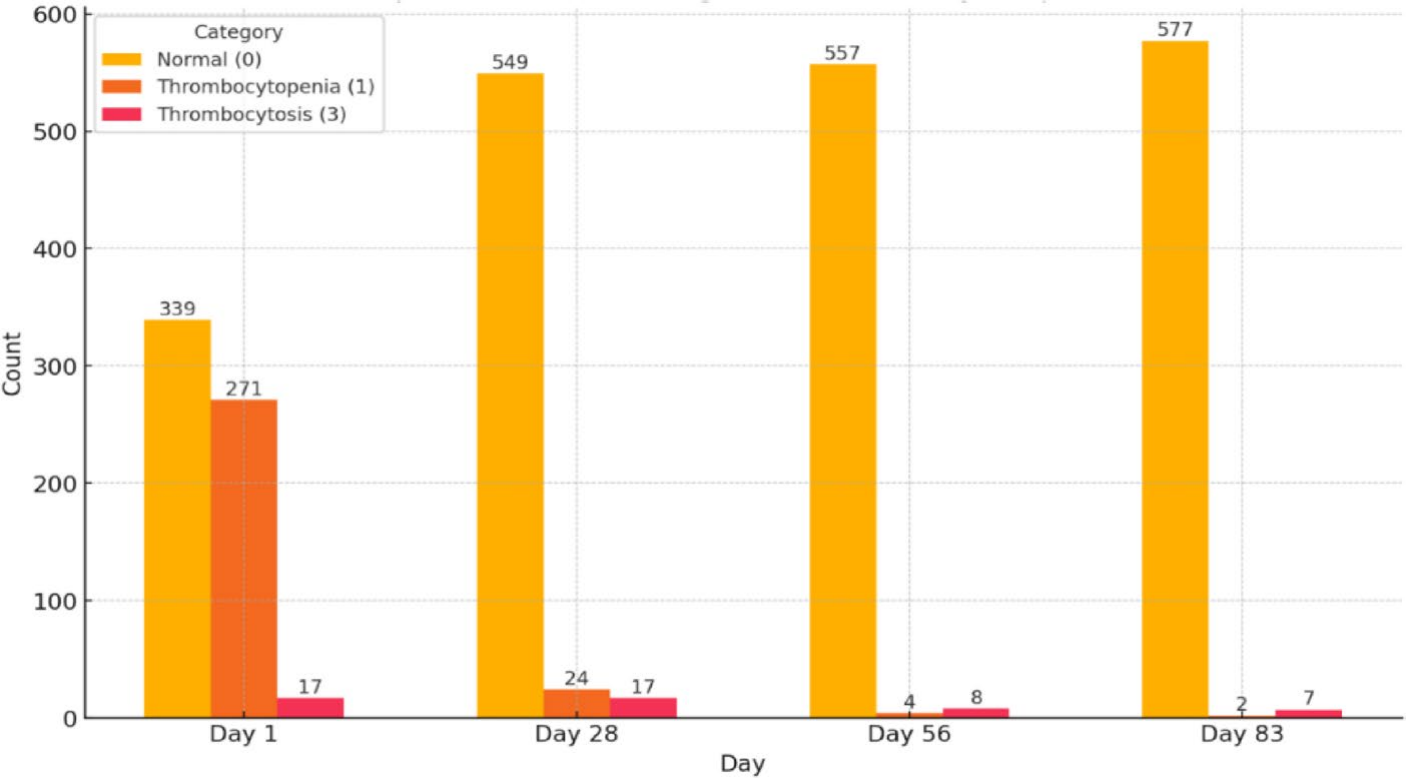
Comparison of platelet categories across treatment time points; Normal range is 175–600 × 10^3^/micL.

**Fig. 5:**
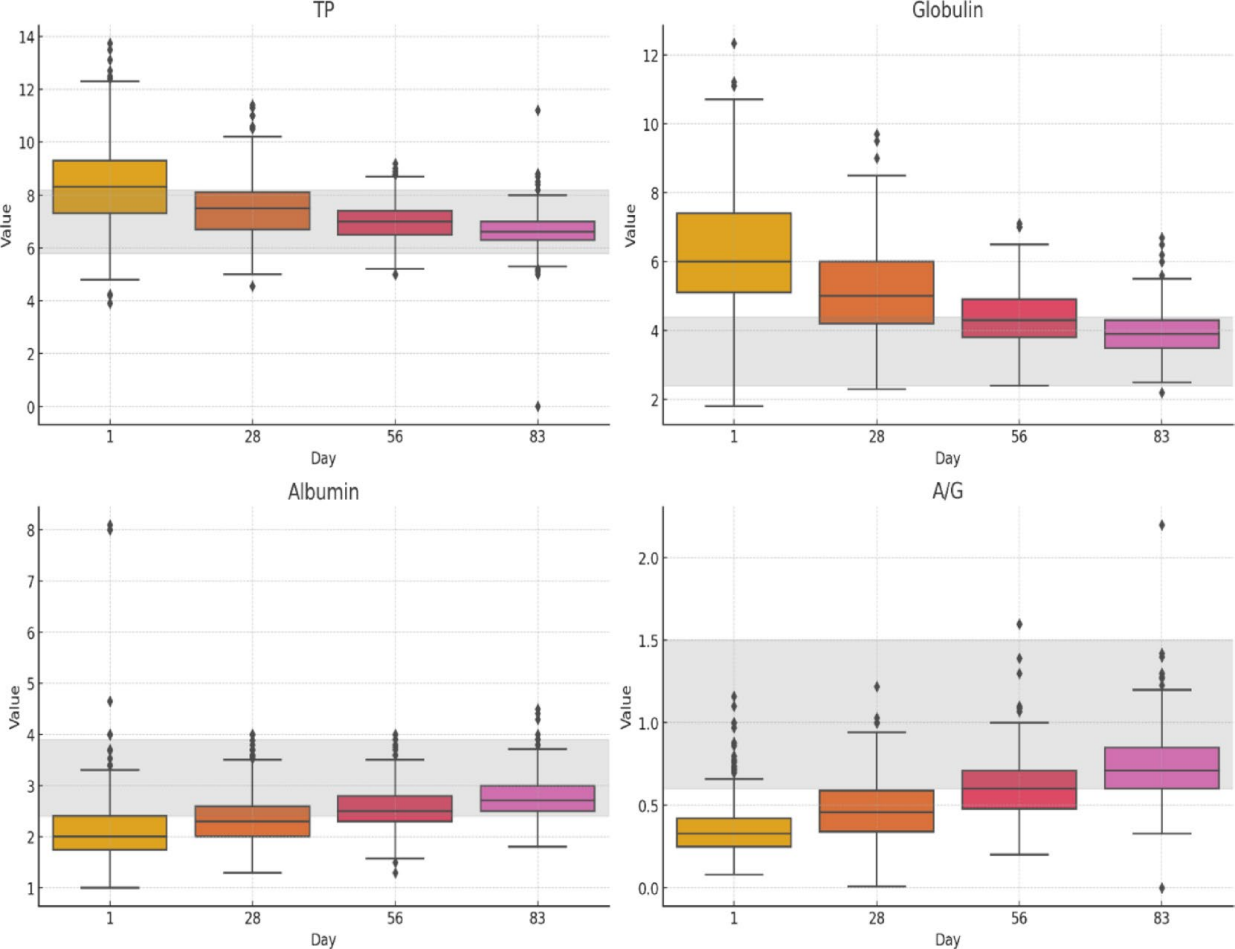
Total protein, globulin, albumin, and A: G ratio level across treatment time points. Normal ranges references are as follows TP, 5.8–8.2 g/dL; Globulin, 2.4–4.4 g/dL; Albumin, 2.4–3.9 g/dL; A/G ratio, 0.6–1.5

**Fig. 6:**
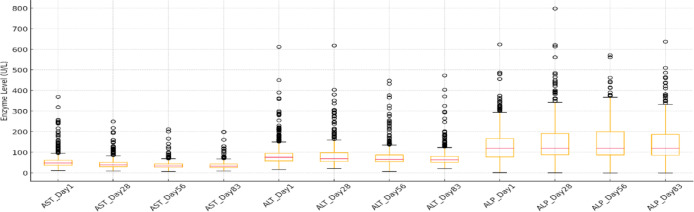
Hepatic enzyme levels across treatment time points. Normal ranges references are as follows: AST 17–48 U/L, ALT 25–97 U/L, ALP 25–93 U/L.

**Fig. 7:**
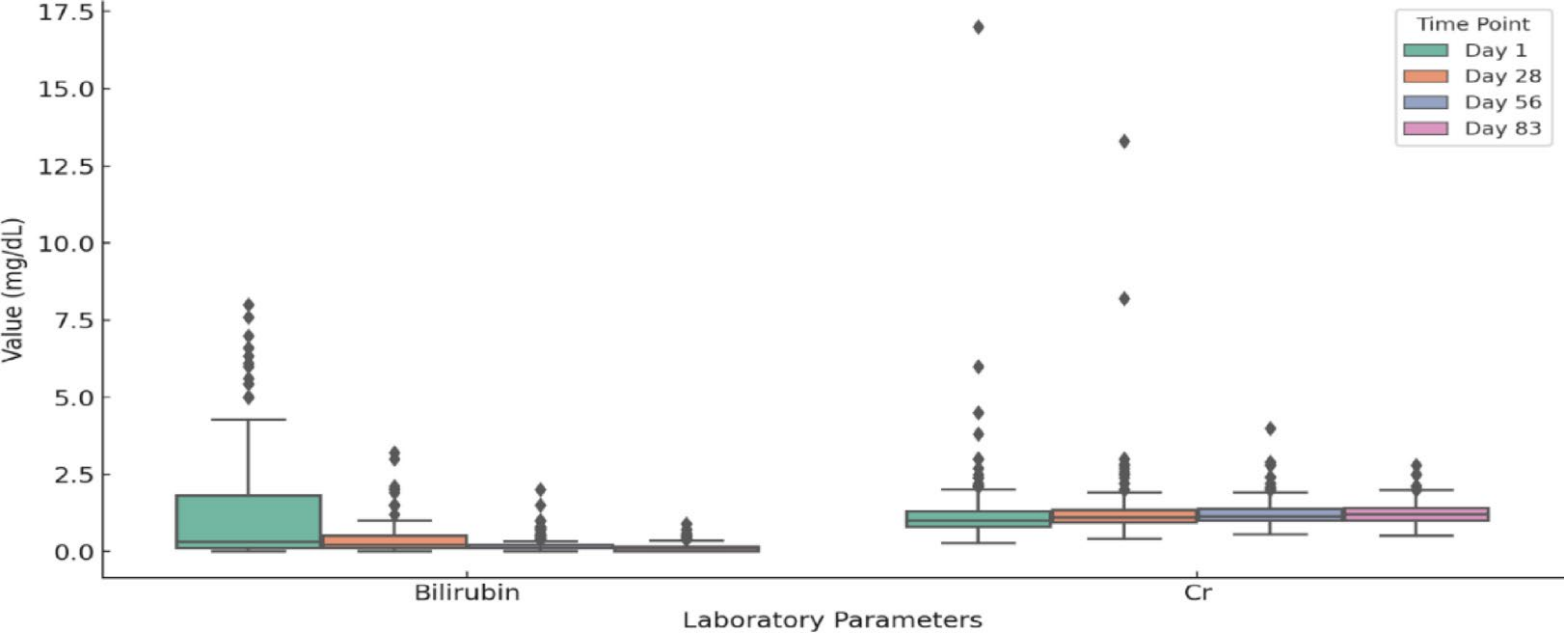
Bilirubin and ceratin levels across treatment time points. Normal range is 0.15–0.5 mg/dL.

**Fig. 8:**
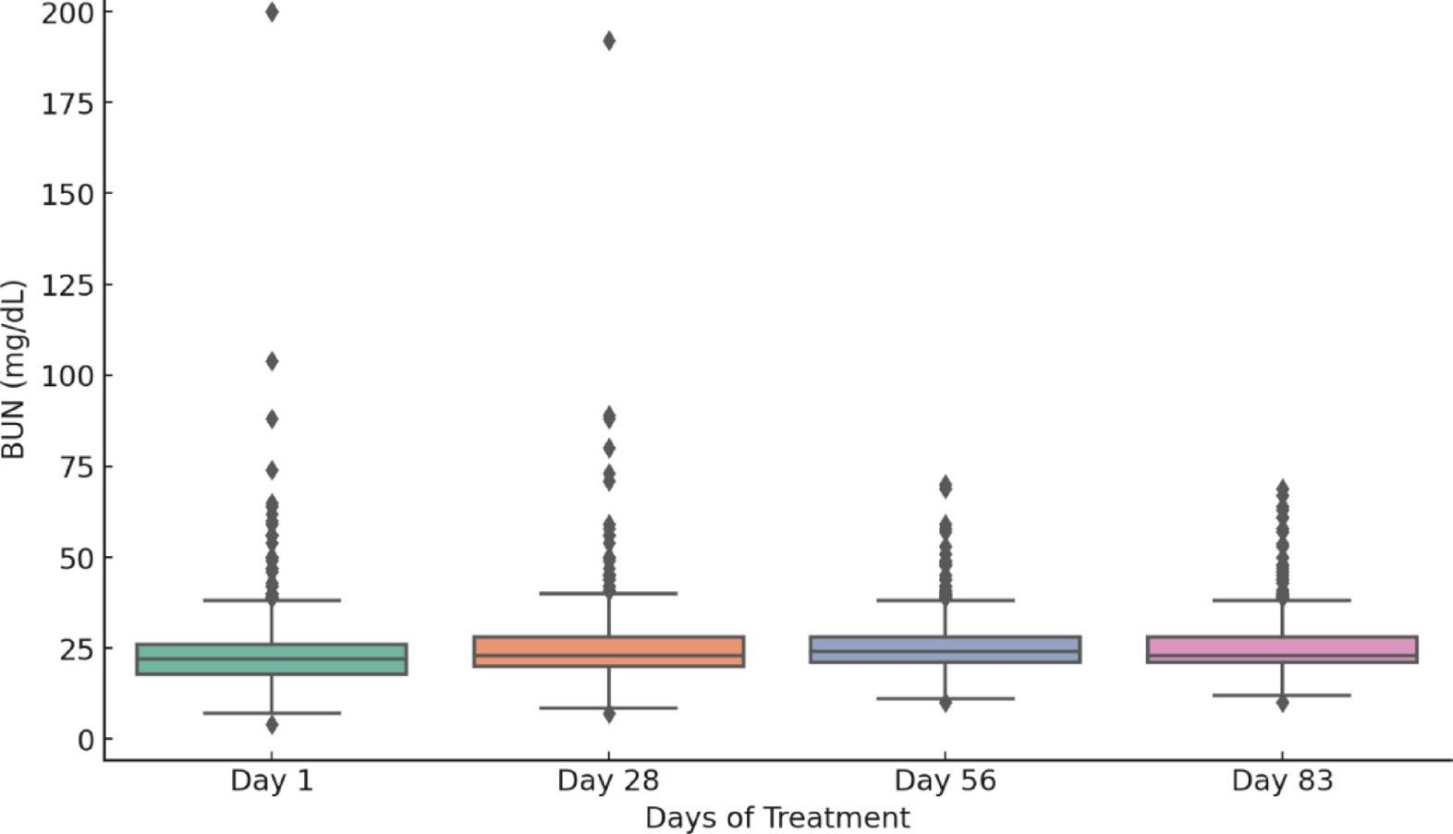
BUN levels across treatment time points. Normal range is 14–36 mg/dL.

Figure 2, 3, 4, 5, 6, 7, 8, and Tables 4 and 5 summarize laboratory findings that change during treatment. Based on Fig. 2, elevated leukocyte counts were observed at first, showing a wide range and numbers outside the normal range. The median leukocyte counts steadily decreased throughout treatment, with narrower interquartile ranges by Day 83, indicating a return to normal values for most cats. In this way, leukocytosis from 32.85% decreased constantly during the treatment to 9.4%, 5%, and 2.7%, respectively, on the 28th, 56th, and 83rd days of treatment. Neutrophil counts showed the leukocyte trend, a notable increase initially. By the conclusion of the treatment, the median neutrophil count had decreased significantly. Regarding lymphocytes on Day 1, 51% of cats displayed low counts; however, by Day 83, only 11.77% demonstrated gradual normalization, indicating higher median counts and less variability. Eosinophil levels remained consistently low throughout the treatment period, with minimal changes observed. Although there was an increase on Day 28 (10.5%) and Day 56 (8.7%) compared to the initial count (6.3%), outliers were few, and median levels remained stable.

**Table 4:** Laboratory findings on Day 83 of treatment with GS-441524 in cats with FIP.

	Count; percentage	Normal range
Anemia (PCV < 24%)	2/586; 0.34%	24–45%
Leukocytosis (Leukocyte > 19,500/micL)	19/586; 3.2%	5500–19,500/micL
Leukocytopenia (Leukocyte < 5500/micL)	35/586; 5.9%	5500–19,500/micL
Lymphopenia (Lymphocyte < 1500/uL)	69/586; 11.77%	1500–7000/uL
Thrombocytopenia (Platelets < 175,000/micL)	2/586; 0.34%	175–600 × 10*3/micL
Increased Total Protein (TP > 8.2 g/dL)	8/586; 1.36%	5.8–8.2 g/dL
Increased Globulin (G > 4.4 g/dL)	120/586; 20.47%	2.4–4.4 g/dL
Decreased Albumin (Alb < 2.4 g/dL)	78/586; 13.31%	2.4–3.9 g/dL
A/G ratio < 0.5	63/586; 10.75%	0.6–1.5
Increased Total Bilirubin (> 0.5)	6/609; 1%	0.15–0.5

**Table 5:** Laboratory findings in the extra treatment time in 63 cats.

	Count; percentage	normal range
Anemia (PCV < 24%)	0/63; 0.0%	24–45%
Lymphopenia (Lymphocyte < 1500/uL)	24/63; 38%	1500–7000/uL
Increased Total Protein (TP > 8.2 g/dL)	2/63; 3.17%	5.8–8.2 g/dL
Increased Globulin (G > 4.4 g/dL)	15/63; 23.8%	2.4–4.4 g/dL
Decreased Albumin (Alb < 2.4 g/dL)	10/63; 15.87%	2.4–3.9 g/dL
A/G ratio < 0.5	8/63; 12.69%	0.6–1.5
Increased Total Bilirubin (> 0.5)	0/6; 0.0%	0.15–0.5

Figure 3 depicts the prevalence of anemia in cats affected by FIP at various treatment intervals. Initially, 363 cats (57.89%) showed signs of anemia, a frequent clinical indicator of FIP. This figure drastically dropped to 31 (5.2%), indicating significant progress during the early phases of treatment, with most cats fully recovering from anemia by day 56. A minor resurgence of anemia was noted in only 2 cats by day 83 of the treatment. Additionally, in 23 cats with effusive forms, it was observed that 10–14 days after starting treatment, despite overall improvement and a decrease in effusion, there was a reduction in their packed cell volume (PCV).

Figure 4 illustrates how platelet categories (Normal, Thrombocytopenia, and Thrombocytosis) are distributed over the treatment period. Initially, 339 cats presented with normal platelet counts, while thrombocytopenia was found in 271 of the 627 cats (43.22%), highlighting a significant occurrence of low platelet counts at the beginning of treatment. A smaller group of 17 cats (2.7%) showed thrombocytosis. Subsequently, the number of cats with normal platelet counts significantly increased to 549, and thrombocytopenia decreased to 4% (24/590) and 0.7% (4/569), respectively, on days 28 and 56. By the end of treatment, 577 cats out of 586 had normal platelet counts, representing the highest percentage of normal counts throughout the treatment period. Thrombocytopenia cats were reduced to 2 cats (0.35%), and thrombocytosis persisted in 7 cats. According to Fig. 5, total protein (TP) levels were elevated (≥ 8.2 g/dl) at the start, with 52.15% of cats exhibiting this condition, showing considerable variability. Over time, the median TP levels gradually decreased, dropping from 52.15% to 22.71% by the 28th day and reaching 3.1% by the 56th day, ultimately approaching ranges by the 83rd day. However, some outliers persisted, suggesting that a subset of cats continued to exhibit persistently high protein levels (1.3%). Elevated globulin levels were observed at the beginning of treatment in 86.92% (545/627) of the cats, which is consistent with the inflammatory characteristics typically associated with FIP. Throughout treatment, globulin levels had a gradual decline. Specifically, levels dropped from 70% (413/590) on day 28 to 45.86% (261/569) by day 56. Additionally, the interquartile ranges decreased from 83% to 20.47% (120/586). This trend indicates a positive response throughout the treatment period. In this study, in 60 cats, total protein (TP) and globulin levels were increased from day 1 to day 28. Of these, 31 cats had effusive abdominal form, 11 were non-effusive, five were effusive thoraco-abdominal, 4 were mixed form, four were neuro-ocular form, and 3 were effusive thoracic. The effusive abdominal and non-effusive forms significantly increased TP and globulin among 60 cats (*p* < 0.01) by the Wilcoxon Signed-Rank Test among all forms. Albumin levels were low at baseline, affecting 73.68% (462 out of 627) of the cats, which likely indicates the hypoalbuminemia commonly associated with FIP. Median albumin levels steadily increased during treatment, with low albumin counts recorded at 56.62% and 28.87% on days 28 and 56, respectively. By the 83rd day, the low albumin count had decreased significantly to just 13.3%. The A/G ratio significantly decreased at the beginning of treatment in 89.47% (561 out of 627) of the cats. The ratio showed improvement as therapy progressed, with median values increasing, indicating a favorable response to the treatment. By day 28, the improvement was noted in 61.35% (362 out of 590), followed by 30.4% (173 out of 569) on day 56, and finally 10.75% (63 out of 586) on day 83. In this study, 65 cats (10.36%) had normal serum protein and A: G ratios at the outset, 51% of which were dry, while eight cats (1.2%) showed hypoproteinemia, and all of them were wet.

In Fig. 6, the analysis of hepatic enzymes was presented. AST was slightly elevated in some cats (282 out of 603) at 46.76%, along with a few outliers. As the study continued across days 28 and 56, the median remained stable, although there was a decrease from the initial values. A trend toward normalization was observed on day 83 when only 11.82% (68 out of 575) of cats were considered outliers. The other enzyme was ALT; some cats initially had high ALT levels (23.21%). On the 28th day, there was a mild increase (25.6%), and the following levels decreased until there was a general decline in ALT at the end, where only 14.95% remained above. ALP levels exhibited the widest variability throughout treatment, with persistently high values and significant outliers at all times. ALP normalization appears less consistent compared to AST and ALT.

The Fig. 7 depicts that bilirubin levels were significantly elevated in some cats, with 261 out of 609 (42.85%) showing high levels initially. However, the median bilirubin levels decreased considerably over time, showing 22.83% (132 out of 578) on day 28 and dropping to 4% (23 out of 561) on day 56. By day 83, most cats exhibited normal bilirubin levels, with only 1% (6 out of 609) still elevated. This Fig indicates that creatinine levels remained stable and low throughout the treatment, suggesting no significant renal compromise during that time. No major shifts were observed from start to finish. The Fig. 8 indicates that BUN levels stayed fairly consistent and within the normal range during the study. Only slight fluctuations were observed, along with a few outliers reflecting mild deviations.

The laboratory abnormalities and changes observed improved by the end of treatment, as summarized in Table 4. Moreover, by the end of the treatment, 63 cats had an A: G ratio of less than 0.5. The treatment for these cats was extended for an additional 10 to 14 days, during which abnormal findings increased once again. Nevertheless, eight cats remained abnormal; all had concurrent diseases (FIV, FeLV, periodontitis).

Table 5 presents the laboratory findings of 63 cats at the end of extra treatment. According to the table, lymphopenia, decreased albumin, increased globulin, and the A: G ratio decreased significantly with additional treatment time, and the low number of cats outlined the normal range values. Meanwhile, anemia and thrombocytopenia are absent, and increased TP occurs infrequently, at only 3.17%.

Statistical analyses were carried out to evaluate the significance of laboratory changes over time. A one-way ANOVA was utilized to compare the A:G ratio, total protein (TP), globulin, and albumin among the treatments. The findings showed a statistically significant difference (*p* < 0.05) over time, suggesting that these parameters had significantly improved.

The chi-square tests were performed to assess whether changes in various parameters during treatment were statistically significant. The results show that the *p*-values for ALP, BUN, and Cr exceeded the significance threshold of 0.05. This finding implies that the observed changes in these parameters throughout the treatment period were not statistically significant. Conversely, the *p*-values for anemia, thrombocytopenia, lymphopenia, leukocytosis, eosinophilia, and bilirubinemia were less than 0.05, confirming a significant reduction in these conditions with treatment. This indicates that the treatment successfully addressed these particular hematological and biochemical issues.

FCoV RNA detection using RT-PCR was done in 88 cats, of which 27 showed negative results despite exhibiting clinical signs of FIP. The test was performed on blood, and 5 of them were on fluids from the abdominal and pleural effusion cavities.

Regarding imaging findings, at the beginning, abdominal ultrasound revealed effusion in 49.35% (308/624) of cats and lymphadenopathy (LN > 5 mm)^22^ in 92.62% (578/624). Hepatic changes, including hepatomegaly and both hypoechoic and hyperechoic echotexture, were observed in 63.14% (394/624). Splenic changes, such as splenomegaly (the mean thickness was more than 1 cm)^22^ and a mottled appearance, were noted in 68.58% (428/624). Renal changes consisted of renomegaly and corticomedullary changes in 23.87% (149/624) as well as a medullary rim sign (an echogenic line in the outer zone of the renal medulla that parallels the corticomedullary junction) in 46.63 (291/624). Gallbladder changes, characterized by a double wall (Gall bladder thickness > 1 mm)^22^ and sludge, were recorded in 47.11% (291/624).

53 (11.25%) and 24 (4.5%) cats, respectively, on 56 and 83 treatment days, reported urinary bladder sedimentation throughout the treatment.

This study presents a summary of abdominal ultrasonographic findings and the changes observed in sonography during the treatment period, illustrated in charts which are explained below.

**Fig. 9:**
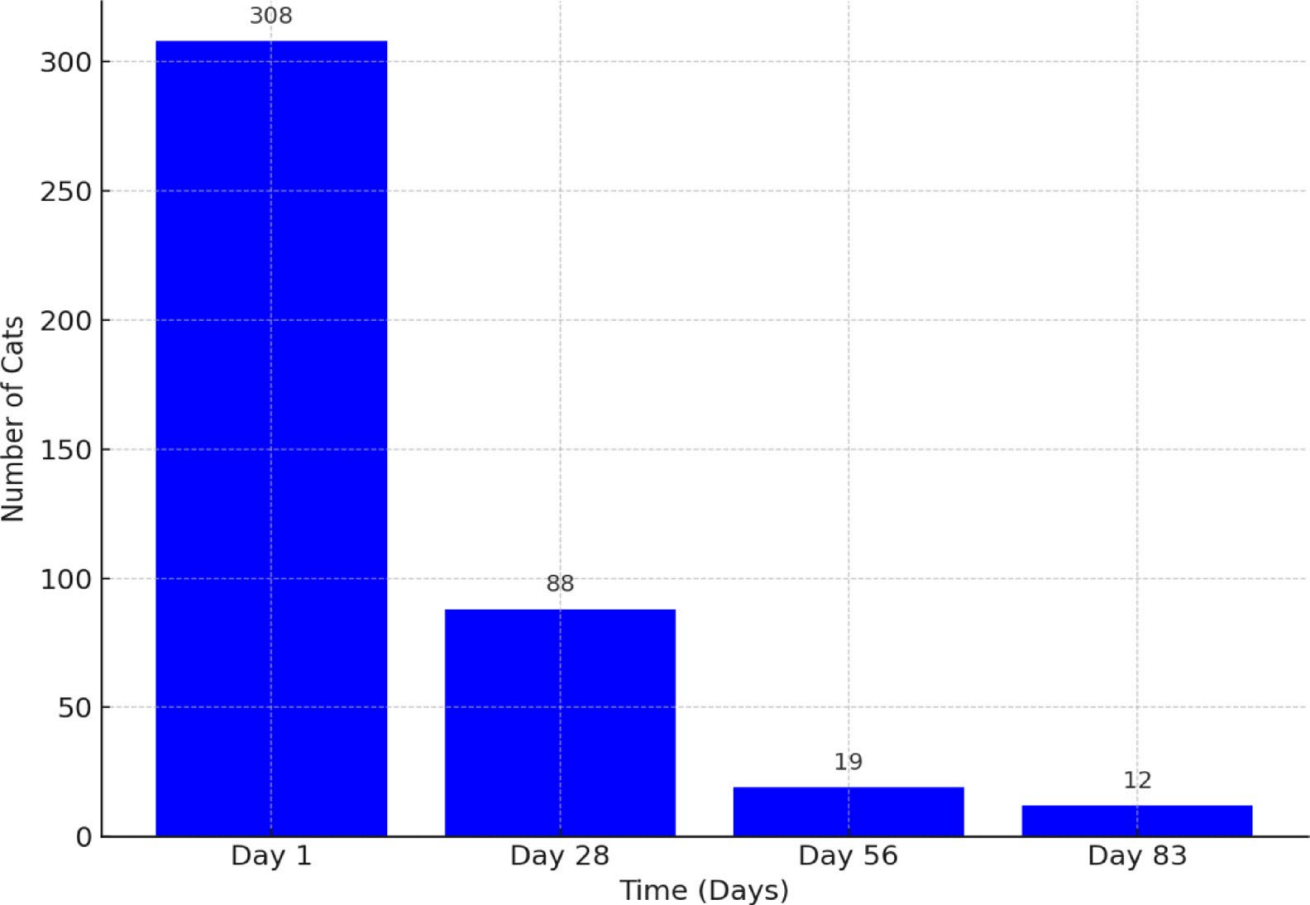
Abdominal free fluid counts across treatment time points.

Figure 9 shows a significant and progressive decline in the presence of abdominal free fluid throughout the treatment period, indicating that the treatment effectively addressed this condition. A total of 88, 19, and 12 cats, respectively, exhibited abdominal effusion on the 28th, 56th, and 83rd days of treatment. In those cats that had effusion at the end of the treatment, extending the treatment with higher doses for an additional 10–14 days was done; however, there was still a small amount remaining in only three cats after following the protocols.

**Fig. 10:**
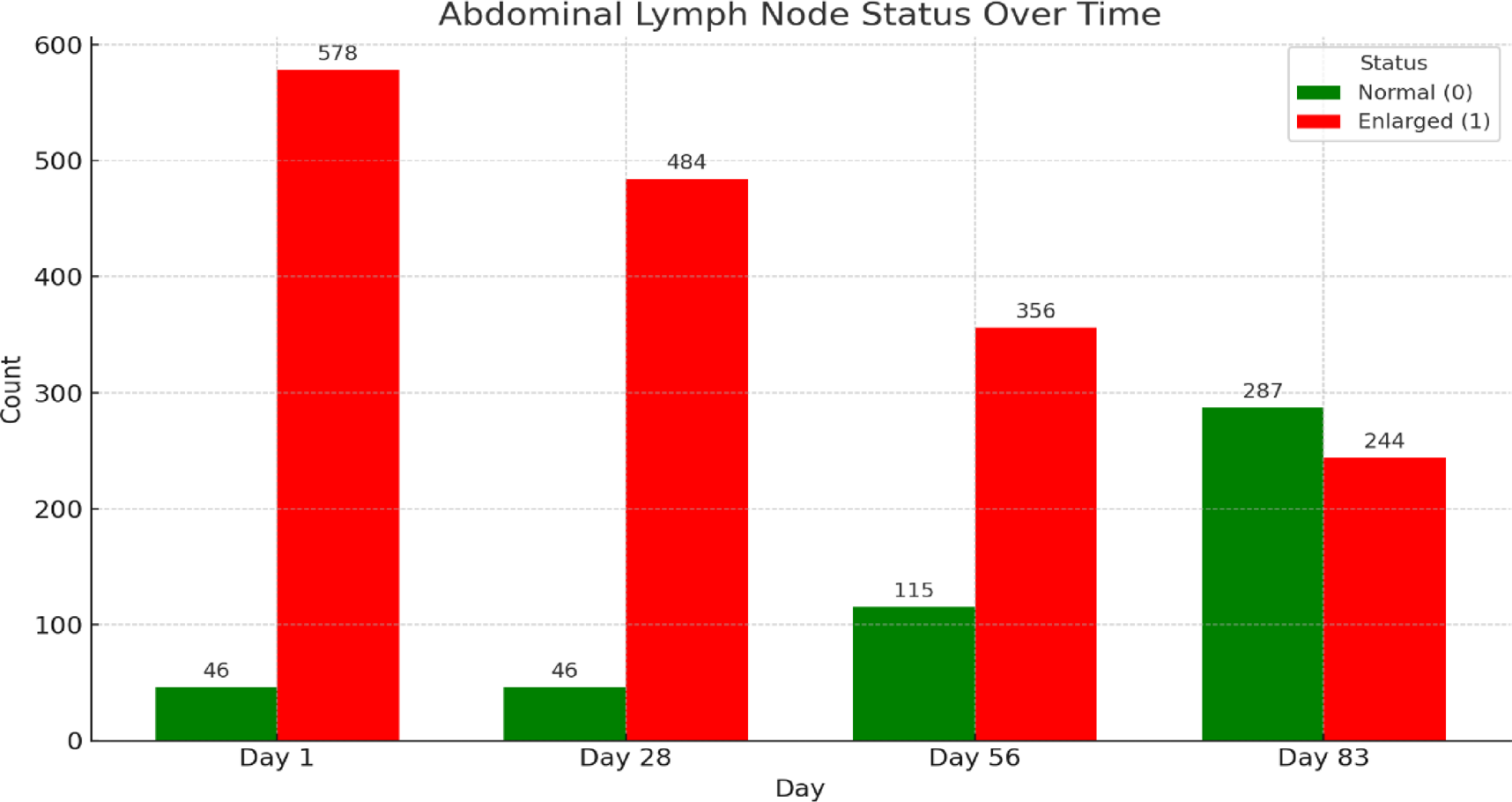
Abdominal lymph nodes status across treatment time points. Lymphadenopathy is defined as lymph nodes with a diameter greater than 5 mm.

Figure 10 trends show a gradual decrease in the prevalence of enlarged lymph nodes over time, alongside an increase in normal cats, which may indicate the treatment’s effectiveness. However, 45.95% of cats (244/531) still exhibited enlarged lymph nodes by the end of the treatment. It is important to note that in all 244 cats, the size of the lymph nodes decreased but did not return to the normal range.

**Fig. 11:**
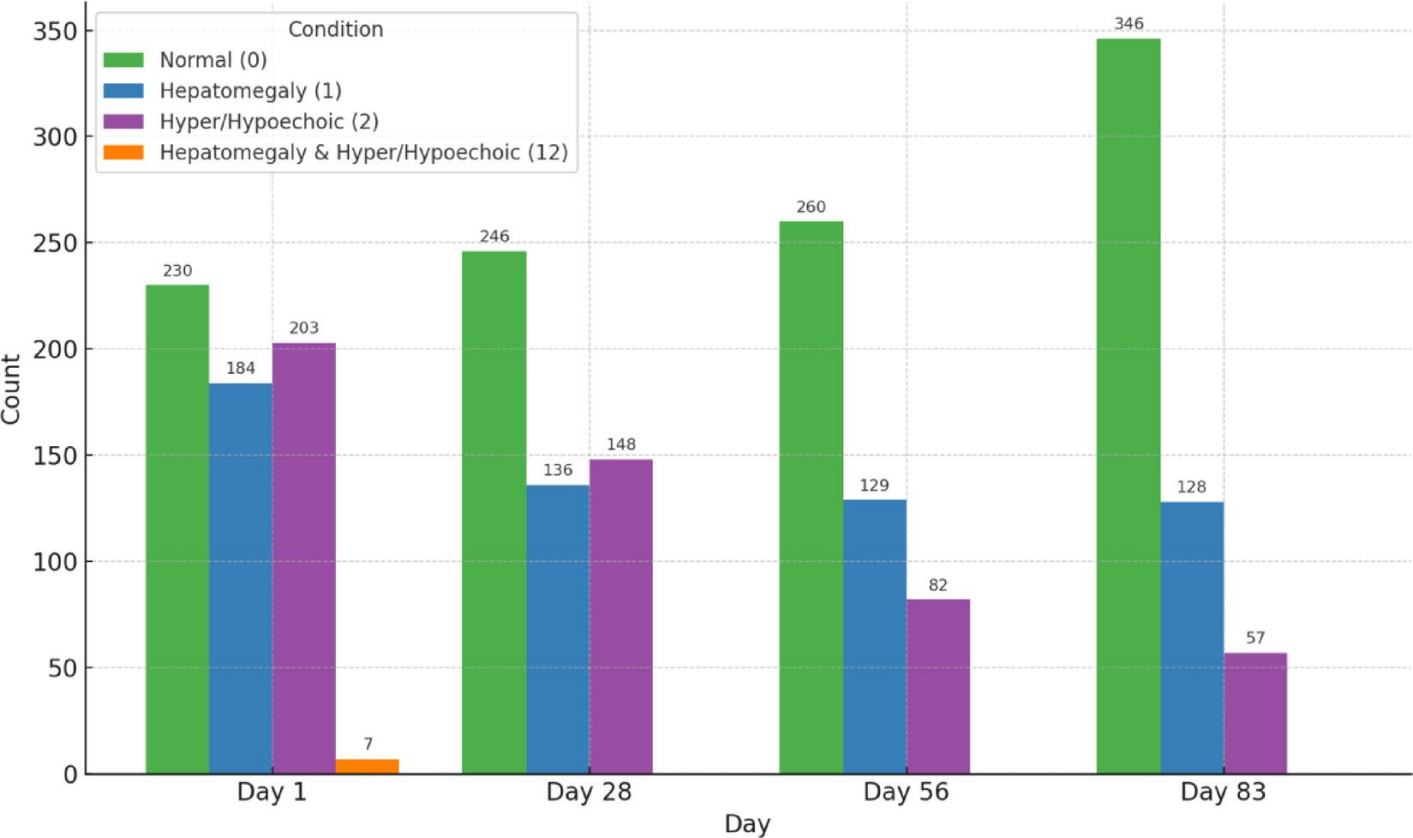
Hepatic changes in sonography across treatment time points.

**Fig. 12:**
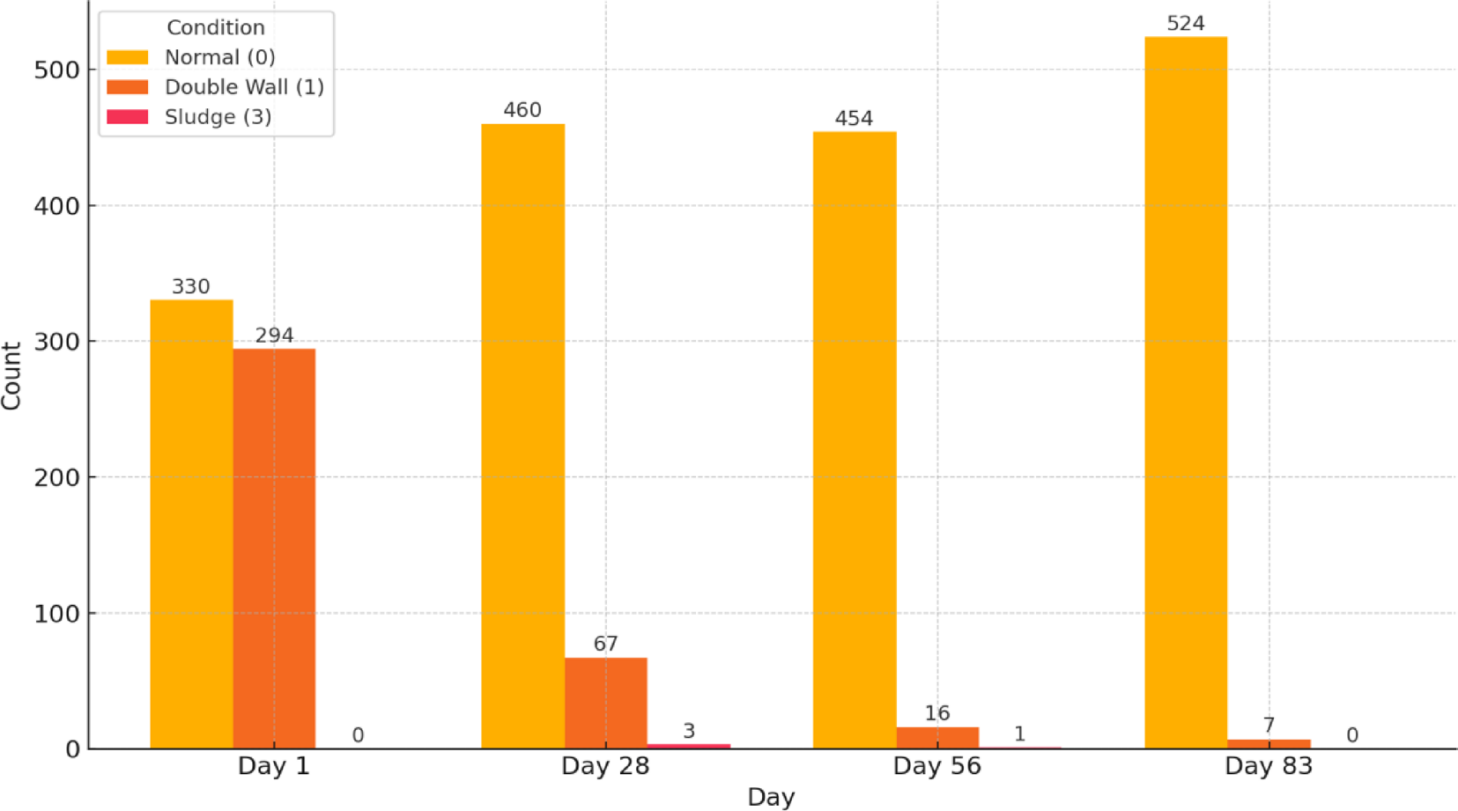
Gall bladder changes in sonography across treatment time points. Increased Gall Bladder thickness is defined as a diameter greater than 1 cm.

Figure 11 shows the hepatic changes observed in sonography throughout the treatment. This progression indicates a consistent improvement in hepatic conditions over time, as reflected by the increasing prevalence of normal sonographic findings from 36.85% (230 out of 624 cats) at the beginning to 68.54% (364 out of 531 cats) at the end. Hepatomegaly and hyper/hypoechoic levels continue to decline over time. The trend Fig. 12 for sonographic findings related to the gallbladder indicates a significant reduction in abnormal sonographic double-wall signs, decreasing from 294 cats (47.11%) at the beginning to just seven cats (1.3%) at the end of the treatment. This observation was not reported in previous studies on FIP cats.

**Fig. 13:**
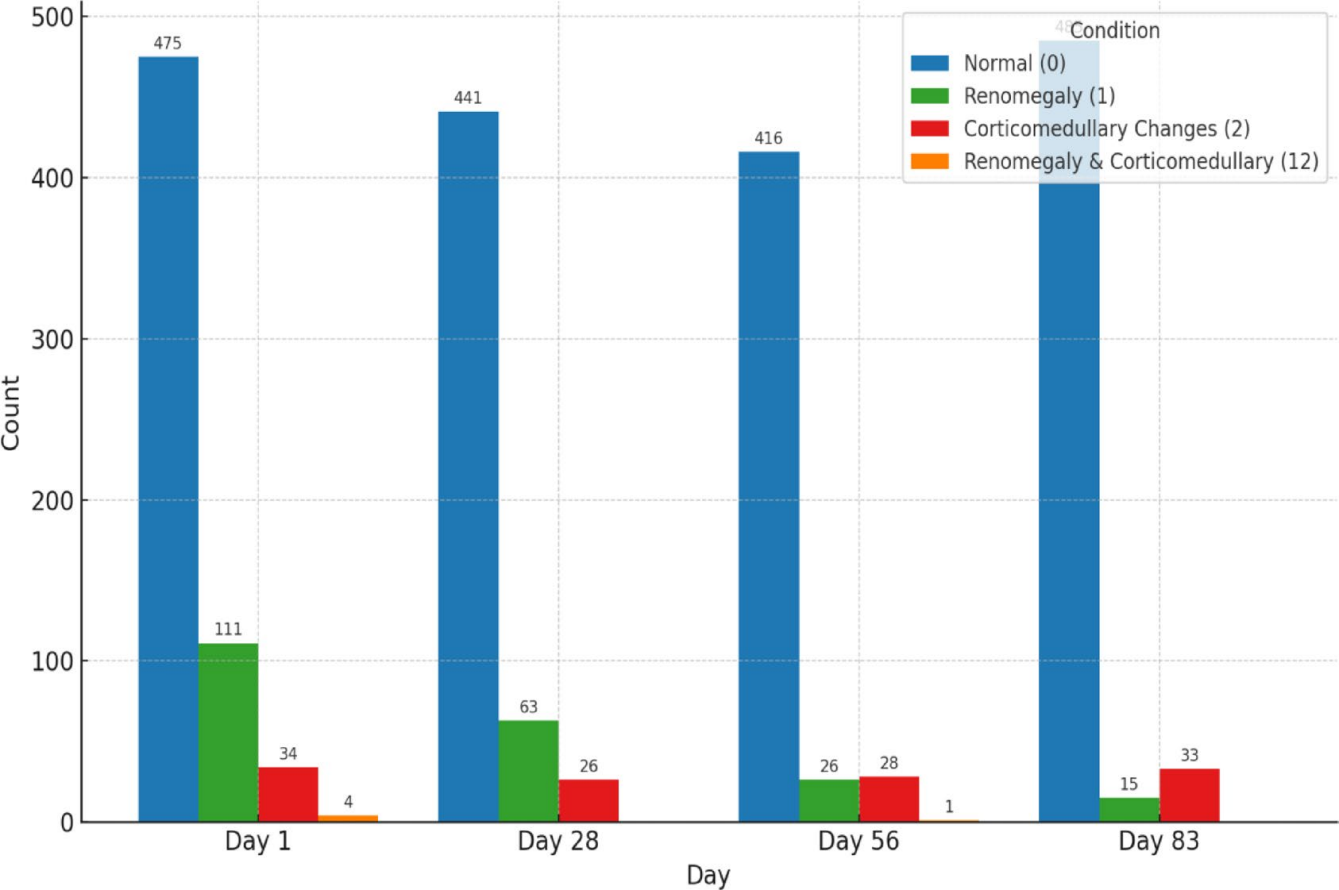
Renal changes in sonography across treatment time points.

**Fig. 14:**
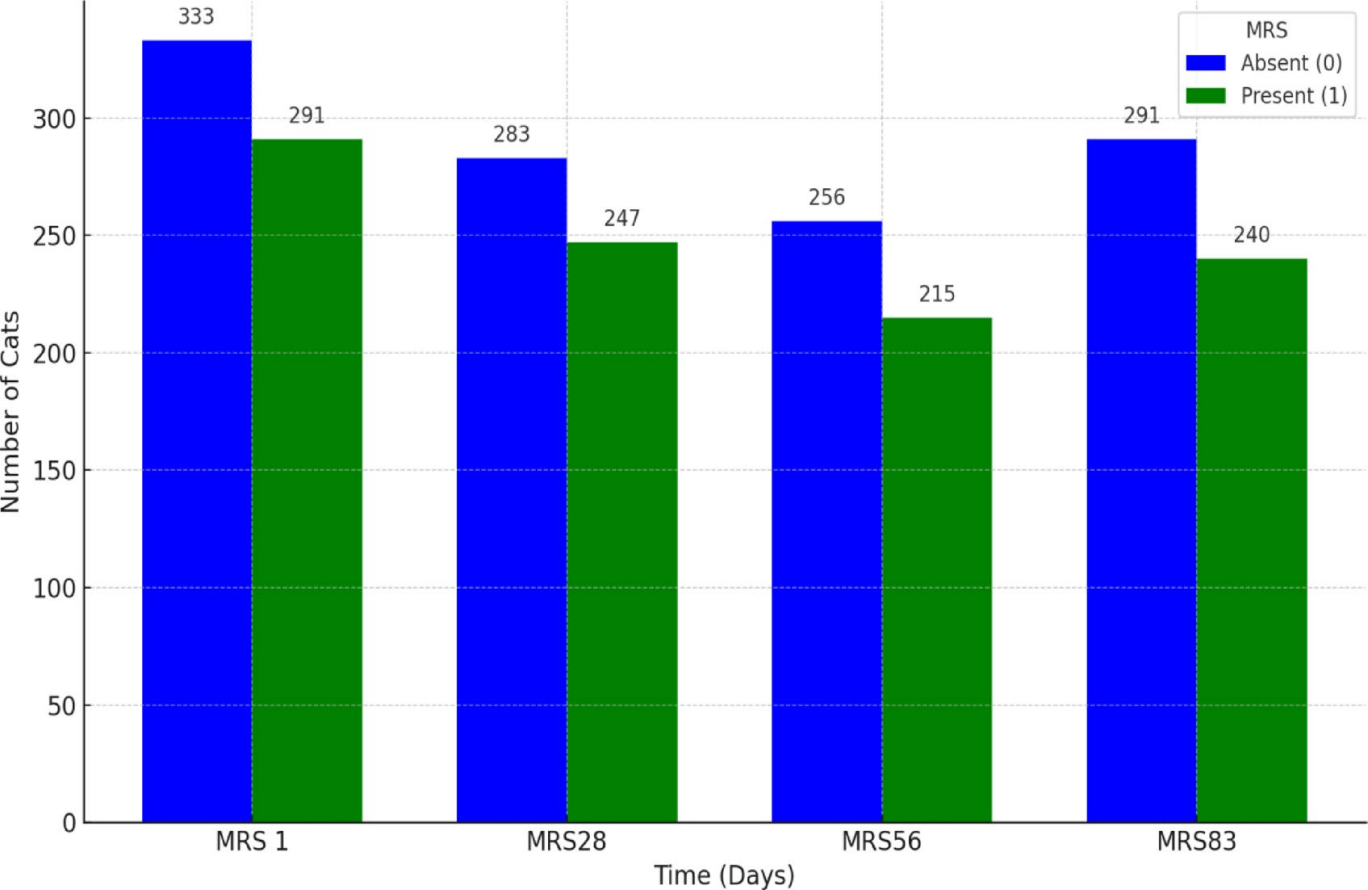
MRS count in renal sonography across treatment time points.

**Fig. 15:**
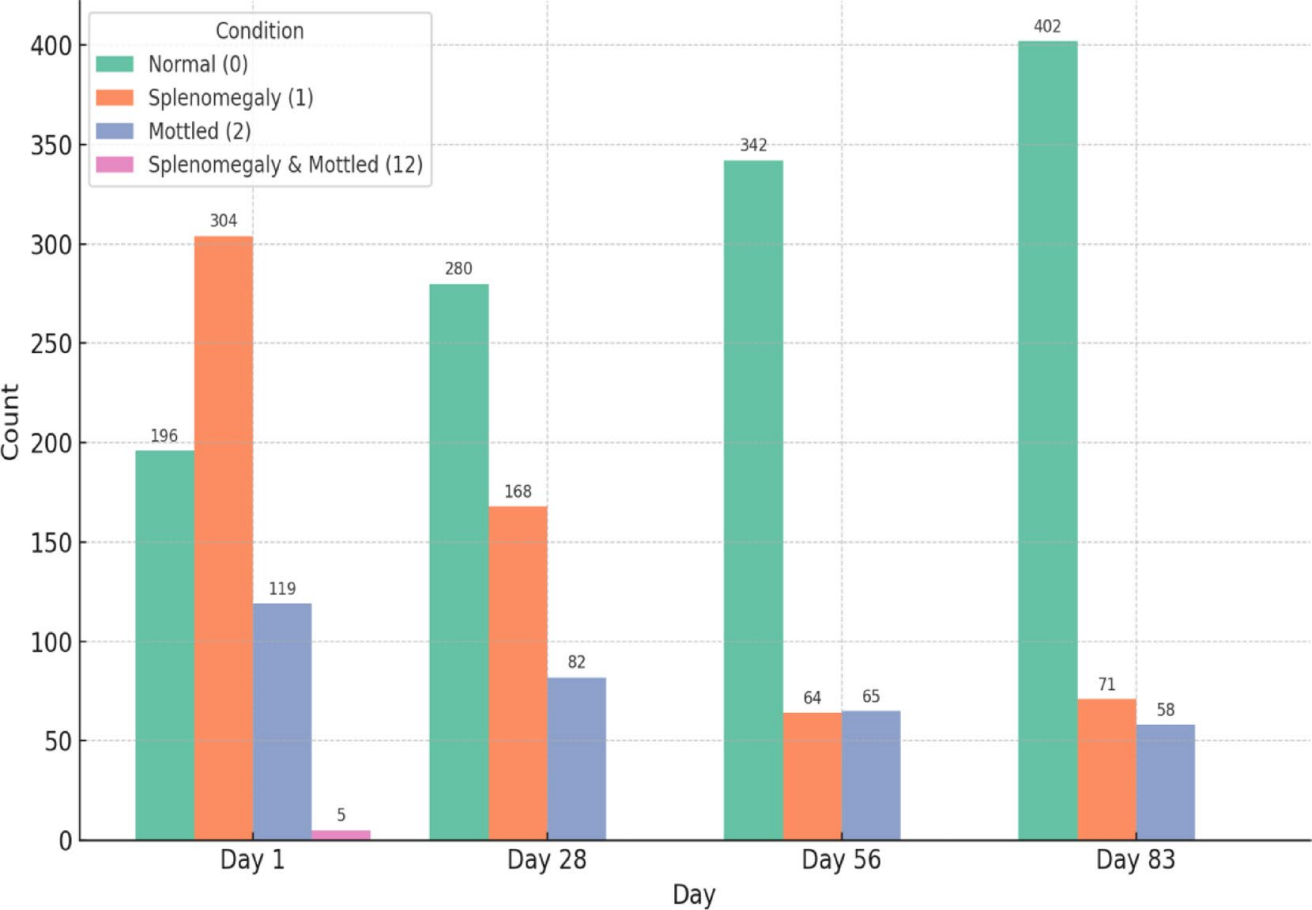
Spleen changes in sonography across treatment time points. Normal thickness was less than 1 cm.

Figures 13 and 14 show the changes in renal conditions observed through sonography during the treatment period. The medullary rim sign (MRS) was reported separately. Excluding MRS, most cats had normal kidneys, and their numbers increased throughout the study, rising from 475 cats (76.12%) at the beginning to 483 cats (90.96%) by the end. Reports of renomegaly decreased from 111 (17.78%) initially to 15 cats (2.82%) by day 83, while corticomedullary changes showed a fluctuating pattern, and the low count did not resolve completely at the end. Meanwhile, the occurrence of MRS remained stable throughout the treatment period, with only minimal fluctuations in its prevalence, which were 46.63%, 46.60%, 45.64%, and 45.19%. Therefore, MRS did not significantly change over time, and treatment did not lead to a statistically or clinically meaningful reduction. Figure 15 shows changes in the spleen observed through sonography during treatment. Initially, splenomegaly was the most common condition (304 cats; 48.71%), followed by normal findings (196 cats;31.41%) and mottled spleens (119 cats;13.37%), with five individuals exhibiting combined conditions. Over time, normal findings gradually increased, while splenomegaly and mottled spleen cases consistently declined. At the end of the treatment, these results indicate a clear trend of improvement in splenic conditions throughout the treatment period.

Crystals were observed in the urinary bladder at rates of 11.25% on the 56th day and 4.5% on the 83rd day of treatment; they were not present at the beginning of the treatment. Additionally, these cats displayed clinical signs related to the presence of crystals.

Chi-square analysis shows statistically significant changes (*p* < 0.05) in all sonographic findings during treatment. However, the medullary rim sign in renal changes is not statistically significant (*p* > 0.05).

A total of 394 cats in the US with abnormal liver findings before starting GS revealed elevated serum AST levels at 49.23% (194/394), ALT levels at 27.91% (110/394), and bilirubin levels at 41.37% (163/394). Among 149 cats in the US with abnormal renal findings prior to beginning GS, 14% (21/149) had elevated serum Cr levels, while 16.1% (24/149) showed elevated BUN levels.

Out of 629 cats, 246 (39.1%) underwent radiographic examinations at the start of treatment. The recorded findings included pleural effusion, enlarged mediastinal lymph nodes, cardiomegaly, a nodular lung pattern, bronchitis, and pneumonia. The observations made during treatment are summarized in Table 6. Initially, pleural effusion was the most common abnormality (89/246 cats), followed by enlarged mediastinal lymph nodes (24/246 cats). A significant improvement was noted with treatment in thoracic radiographic conditions, as regression in these two common findings occurred in all affected cats by the end of treatment (Table 6).

**Table 6:** Summary radiographic findings across treatment time points.

Day	Count	Pleural effusion	Enlarged mediastinal LN	Normal	Cardiomegaly	Bronchitis	Pneumonia	Nodular lung pattern
0	246	89	24	118	34	17	4	2
28	89	5	23	32	31	11	2	0
56	38	0	0	17	17	9	0	0
83	44	0	0	14	24	5	1	0

MRI abnormalities in 3 cats include ependymal contrast enhancement, ventriculomegaly, and meningeal contrast enhancement. 2 of them had hydrocephalus.

The mean starting dosage for effusive forms was 6.9 (6–8 mg/kg), with a standard deviation and median of 0.87; the mean ending dosage was 10.11, with a standard deviation and a median of 1.59. A paired t-test showed that the difference between the starting and ending dosages was highly statistically significant (*p* < 0.05). The mean starting dosage for non-effusive forms (dry, neurologic, and ocular types) and mixed forms was 9.65 (ranging from 9 to 10 mg/kg), with a standard deviation and median of 0.54. The mean ending dosage was 12.7, with a standard deviation and median of 1.15. The paired t-test results are for the non-effusive and mixed forms; the *p*-value is highly small (much less than the standard of 0.05), indicating that the difference between the starting and ending dosages is statistically significant. Specifically, the mean starting dosage for the neurologic form was 10.02 mg/kg, compared to 6.9 mg/kg for the effusive form. At the end of treatment, the mean dosages changed to 13.23 mg/kg for the neurologic form compared to 10.11 mg/kg for the effusive form. These differences were found to be statistically significant.

In this study, out of 259 cats with neuro and/or ocular signs (note that the mixed form is included), 239 cats were cured, and two had relapsed and been treated again. One of them died five months after the second treatment due to sepsis and internal complications of parasites. In conclusion, nine cats exhibited mild neurological symptoms, including urinary incontinence, difficulty urinating, mild ataxia, and seizures. Additionally, four cats displayed ocular symptoms such as increased intraocular pressure (IOP), uveitis, and blindness. These symptoms persisted even after increasing the dosage and treatment duration beyond 84 days.

In the current phase of publication, the following outcomes have been reported:

- 592 cats (94.12%) were cured (survival rate).
- None of the cats in this study were in the observation period (having survived at least 12 weeks of treatment) at the time of the article’s presentation.
- 4 cats (0.63%) were undergoing treatment again for a relapse of FIP signs.
- A total of 37 cats (5.88%) died during treatment; 33 of them passed away before the 20th day, while 4 died later.
- 4 cats (0.63%) died following the observational period.
- The total number of deaths is 41 cats; the overall mortality rate is 6.48%.
- Given the four monitoring factors related to treatment, the duration was extended beyond 84 days for 109 cats (17.32%).
- 5 cats (0.8%) completed a treatment period shorter than 84 days, and all of them are still alive.

The ANOVA test determined whether treatment duration varies significantly among different FIP forms. The results show that the *p*-value is greater than 0.05, suggesting no statistically significant difference in treatment duration across the various FIP forms.

Statistical analysis employing a t-test identified multiple prognostic factors influencing survival outcomes. Male cats had reduced survival rates compared to females (*p* = 0.0483). Cats aged 12 months or younger exhibited the highest survival rates, whereas those older than 6 years had the lowest (*p* = 0.0000892). 3. The effusive form showed a high survival rate, whereas neurological and mixed FIP had the poorest outcomes (*p* < 0.05). 4. Clinical and laboratory findings: Significant risk factors contributing to decreased survival rates included fever (*p* = 0.0038), icterus (*p* = 0.0029), anemia (*p* = 0.0142), and thrombocytopenia (*p* < 0.05). Conversely, factors such as breed, household type, indoor or outdoor conditions, and coexisting diseases had no significant relationship with survival, as evidenced by *p*-values greater than 0.05 in t-tests.

Of the 629 cats, 497 (79%) received injections during the treatment period, while only 132 cats (21%) were treated with a combination of both oral and injectable treatments.

A total of 95 cats underwent surgery during their treatment: 91 cats received ovariohysterectomy, castration, and dental care, while the remaining surgeries were for emergency situations such as intestinal intussusception and limb fractures.

In the follow-up assessment conducted the day after finishing treatment, the cats’ survival duration ranged from a minimum of 300 days to a maximum of 1,470 days.

## Discussion

This survey reports the first use of GS-441524 to treat FIP in cats in Iran, highlights the treatment of the largest known cohort of FIP-affected cats worldwide, and provides a comprehensive evaluation of clinical, laboratory, and imaging changes in cats undergoing treatment with GS-441524. The primary objective is to present this information in a way that allows veterinarians to access a standardized treatment protocol and improve their understanding of all therapeutic aspects of GS-441524, including key factors influencing survival rates for treatment in cats with FIP.

The majority of cats in this study originated from Tehran, the capital of Iran. This distribution is likely due to the author’s affiliation with veterinary centers in Tehran, where referral rates for FIP are higher and diagnostic facilities are more readily available compared to other cities. In this study, 412 male cats (65.5%) were diagnosed more frequently than female cats, consistent with the findings of Sara Jones et al. and Rohrbach et al. Riemer et al.^17,23,24^. Also demonstrated a significant correlation between male sex and FIP prevalence. In this study, data revealed that a significant majority of the cats, 76.31%, or more than three-quarters, were diagnosed with FIP before reaching one year of age, with fewer cats occurring in older age groups; this aligns with findings from multiple studies have reported the highest prevalence of FIP in young cats^1,23,25–28^.

A retrospective study by Pesteanu-Somogyi in 2005 found that purebred cats were significantly more likely to be diagnosed with FIP^29^. Additionally, some studies have reported that FIP is more common in purebred cats^24,25^. This study indicated that DSH cats comprise more than 60% of the population, which likely reflects the overall high proportion of DSH cats in Iran. The purebred Scottish was the second most frequent breed, making up almost one-fifth of the population. Based on the author’s clinical observations, male Scottish kittens appear particularly sensitive to FIP.

FIP is more prevalent among indoor cats in multi-cat households^25,30^. In shelters where multiple cats reside, the overall incidence of coronavirus shedding and the percentage of chronic coronavirus shedders are higher than in single-cat households^31^. This study also showed that the disease was more common in indoor cats and among households with several cats. In these circumstances, the primary factor contributing to increased FIP is the shedding of FECV in the feces of numerous healthy cats within extensive multi-cat settings^32^. When FECV replication is particularly high, various types of mutants of FECV that may lead to FIP are likely to emerge^33,34^. One of the risk factors for developing this disease is stress^25^. In this study, stressful situations, including vaccination, surgery, moving, introducing new members, and grooming, were documented in cats for which information was available (583 cats) within one month before symptoms appeared. Nearly half of them (48.54%; 283 cats) had experienced the mentioned conditions. Pedersen (1995) noted that bringing new cats into the cattery posed the most significant risk of spreading the disease on the premises^1^. Stressful events are considered major contributors to the development of FIP, likely because of their effects on the immune system and viral replication^28^. Additional stressors may include diseases recorded in this survey, such as FPV, URD, and diarrhea. This aligns with earlier findings, highlighting the importance of reducing stress in feline populations to decrease the occurrence of FIP, particularly in high-risk environments like catteries. Furthermore, investigating the background of stressors in cats can lead to a gaudiness for diagnosing FIP. Data indicates that approximately 75% (471 out of 629) of the surveyed cats with FIP also had other illnesses, particularly infectious and systemic conditions. These diseases may have existed or developed during the FIP treatment process. This highlights the complex clinical presentations and treatments that can arise in cats with FIP, which must be addressed concurrently with therapy GS-441524 to ensure a successful response to FIP treatment. Most commonly reported infections included Mycoplasma haemophilus and URD, with toxoplasmosis, FeLV, and FIV in continuation. These infections can influence clinical presentations, such as anemia, infectious, neurological issues, and symptoms of ocular conditions. Previous studies have shown that co-infection with immunosuppressive viruses, such as FIV or FeLV, compromises immune function and possibly worsens the prognosis and treatment of FIP. In fact, they may elevate the risk of FIP development in FCoV-infected cats^35–37^. A notable observation in this study was that those who did not achieve a normal A: G ratio among cats needing prolonged treatment (more than 84 days) were consistently positive for concurrent diseases. As reduced A: G ratio is a well-known diagnostic marker for FIP^38^, based on these findings, persistent A:G ratio abnormalities may indicate underlying infections or systemic conditions that hinder recovery and prolong treatment duration. In this survey, effusive forms were most prevalent (345/629; 54.84%), as in other studies^17,28,39^. The prevalence of effusive forms is comparable to that found in the literature currently in publication because these forms of FIP are easier to diagnose because of their distinctive clinical signs, such as fluid accumulation, which can be identified by physical examination or imaging results and may be subjected to cytological examination and analysis^2,27,40,41^. Treatment results may be impacted by the timely beginning of antiviral therapy, which is probably facilitated by the early diagnosis of peritoneal or pleural effusion. On the other hand, 284 out of 629 cats, or 45.15% of the total, were neurological/ocular and non-effusive types that did not accumulate fluid in this study. This form’s more modest and changeable clinical presentation indications contribute significantly to the difficulties in diagnosing it^18,42^. One-third (190 out of 284) exhibited clinically significant findings, demonstrating FIP’s tendency to affect both the central nervous system and the eyes, often resulting in severe and complex presentations^18^ that require further investigation for accurate diagnosis and management. Categorizing the FIP types in this survey is crucial for demonstrating optimal dosage management for each group. As effusive forms responded well to standard treatment regimens, while neurological and ocular presentations required higher GS-441524 dosages due to the blood brain barrier (BBB) limiting drug efficacy penetration and presenting high population-affected neurological cats, it is also important for examining how they respond to this protocol and how they exhibit long-term survival follow-up^18^.

According to Yin et al.^12^ and Taylor et al.^28^ and this study lethargy (95.2%), decreased appetite or inappetence (91.1%), weight loss (85.1%), and fever (80.2%) were the prominent clinical symptoms that not only all of them are nonspecific for FIP but also correspond to the systemic and inflammatory processes of the disease. Consequently, they should be considered besides medical history, laboratory, and imaging results. Nonetheless, early detection and evaluation of therapy response depend on these clinical indicators, and their reduction may indicate a favorable treatment outcome. In particular, the diagnosis of dry FIP may rely on the therapeutic response (Taylor et al.^12^) which was also assessed in this survey. An interesting observation in this study was the gnashing behavior reported in 11 cats, which has not been documented before. Owners noted that their cats made a noise and had difficulty eating as they moved their teeth against each other despite no apparent issues in the mouth, such as periodontitis or ulcers. With treatment, this sign was eliminated completely. It could be considered a neurological sign. The median duration of clinical symptoms prior to diagnosis of FIP was 14 days, ranging from 5 to 190 days. In another studies, the times were 10 (1–210) days and 12 months (3–96 months)^12,43^. This variation highlights the different rates of disease progression in cats with FIP; as in acute forms, symptoms generally manifest with a rapid onset, while in chronic forms, especially the dry form, they may present more gradually, thereby delaying early detection and intervention efforts. This often results in a poor prognosis with lasting and irreversible neurological or visual complications; therefore, there is a need for rapid identification and initiation of treatment, as prompt initiation of antiviral therapy can enhance survival and reduce the risk of long-term complications. Signalment and clinical findings, along with classic indirect tests (CBC, total serum albumin and globulin levels, A: G ratio, and basic blood chemistries), typically establish the diagnosis of FIP^16,35,44,45^. The prominent abnormalities considered target parameters for diagnosing FIP include anemia, increased serum protein levels (especially characterized by higher globulin and lower albumin), a reduced albumin-to-globulin (A: G) ratio, and hyperbilirubinemia. These results align with the inflammatory and immune-mediated pathological mechanisms commonly observed in FIP, serving as vital indicators of the disease’s severity^16,39,40^. Furthermore, these findings provide necessary details on the course of illness and the efficacy of therapy. The study reveals significant improvements in hematological and biochemical markers in cats diagnosed with FIP who were treated with GS-441524, underscoring the effectiveness of the antiviral therapy. Moreover, even if some FIP-affected cats display normal laboratory findings, depending only on these criteria might lead to a missed diagnosis. For instance, the A: G ratio is often touted as a useful predictor of FIP infection. In our study, 10.36% had normal serum protein and albumin to globulin (A: G) ratios at the outset. Among these, 51% were in dry form. Meanwhile, 1.2% exhibited hypoproteinemia, all of which were in the wet form. The reasons could be explained in the effusive (wet) form of FIP, significant amounts of protein-rich fluid are lost into the body cavities during vasculitis. However, this effusion is typically high in protein. Large-volume effusions may deplete serum protein levels over time, particularly if protein loss exceeds hepatic synthesis besides it the protein loss in cats with FIP is caused by glomerulopathy secondary to immune complex deposition, by loss of protein due to exudative enteropathy in the case of granulomatous changes in the intestines^2^. The effusive abdominal and non-effusive forms were statistically significant in those cats that showed increasing TP and Glu from day 1 to 28. These modifications likely indicate the evolving immunopathogenesis and systemic inflammatory response typical of FIP^38,46^. This disease, particularly in its effusive and non-effusive forms, induces a chronic immune response dominated by humoral activity. Polyclonal B-cell activation results in excessive immunoglobulin production and hyperglobulinemia^16,38,46,47^. This may be why serum globulin concentration showed the most extended normalization among all monitored parameters during treatment, as in the study by Tayloe^12^. Furthermore, persistent antigenic stimulation and prolonged antigen exposure due to ongoing feline coronavirus replication within monocytes and macrophages contribute to sustained immune activation^34^. This underscores the necessity of considering their history, signalment, physical examination, imaging findings, and laboratory results. In some cats with wet form in this survey, after initiating treatment and despite showing a response with regression of fluid and weight loss, along with signs of recovery during the physical examination, there was a drop in packed cell volume (PCV). This could be attributed to fluid resorption back into circulation, which is not necessarily a bad sign.

The survey started by collecting imaging data as a diagnostic tool. However, the author and other researchers (Pedersen^16^) found that the imaging results are not specific to FIP and should be evaluated alongside clinical history and physical abnormalities. Nevertheless, with its larger population, this study offers more comprehensive information on imaging changes in cats with FIP and how they progress during treatment. For instance, according to the results of this study, less than 50% and 20% of affected cats, respectively, show abnormal serum biochemical markers related to hepatic and renal changes. Also, all infected cats with changes in their hepatic and renal ultrasonography did not show abnormal results in the laboratory. Therefore, the importance of combining serum chemical analysis with imaging evaluation for diagnosis and treatment monitoring is emphasized. In this study, abdominal effusion was the most common ultrasonographic finding, consistent with Müller’s^22^ findings, with 88% of cats presenting this feature. Abdominal effusion was a critical parameter in evaluating the therapeutic efficacy of GS-441524 for treating wet or mixed forms of FIP. Based on this study, regular ultrasound monitoring is essential to identify effusion and assess treatment progress and responses. The complete resolution of effusion serves as a crucial index of the effectiveness of therapy and long-term recovery. The ongoing presence of abdominal effusion in a small number of cats highlights the necessity for continuous monitoring and potentially extended or adjusted treatment protocols to achieve favorable outcomes. Additionally, abdominal lymphadenopathy is a notable feature of FIP, particularly in non-effusive forms, as previously noted by Kipar^47^ and Müller^22^. In the current study, 92.62% (578/624) of cats exhibited abdominal lymphadenopathy, which is significantly higher than previous findings investigation^22^. While a significant reduction in lymph node size was observed during treatment with GS-441524 (*p*-value < 0.05), 25% of cats still exhibited lymphadenopathy (LN > 5 mm) at the end of therapy. Notably, in these cats, where clinical signs, physical examination, laboratory findings, and weight gain were normal, treatment discontinuation was successful, with no recurrence of FIP during follow-up. This suggests that residual lymphadenopathy may not indicate active disease in all cats despite the reduction in size compared to the previous one, which is important. However, persistent lymphadenopathy accompanied by abnormal clinical or laboratory findings may warrant extended therapy or further investigation, as these cases are at higher risk of relapse than one of the relapsed cats in this study had this situation. Additionally, it is critical to recognize that abdominal lymphadenopathy is not specific to FIP. Differential diagnoses, including round cell neoplasia, reactive lymphadenomegaly, and other infectious diseases (bacterial, fungal, or protozoal), should always be considered when evaluating cats with this condition^22^. One of the internal organs frequently affected by FIP is the liver^48^. In this study, the findings of hepatic changes in the US are 63.14% (394/624), including hepatomegaly and hypoechoic or hyperechoic echotexture. Besides hepatic changes, this survey reported a double-wall gall bladder or gall bladder wall edema (GBWE) 47.11% (294/624). Although this change is not specific in the US of FIP cats but, hypoproteinemia, and immune-mediated anemia are the reasons for its occurrence^49,50^, and both could happen in FIP cats and have not been reported in previous studies. Non-specific US findings for the spleen in this research, 68.58% (428/624), are similar to those reported in previous studies, including splenomegaly and mottled spleen^22,51^. Extramedullary hematopoiesis, passive congestion, and round cell neoplasia are differential diagnoses for mottled spleen appearance^47^. The kidney is the organ most susceptible to FIP-related conditions, which would suggest the presence of non-effusive FIP lesions. Gülersoy et al. reported that renal ultrasonography could provide useful clinical information in evaluating the clinical reflection of vasculitis due to FIP^52^. Renal changes, including renomegaly, corticomedullary changes, and medullary rim signs, were recorded in this study. Among these, the MRS was approximately consistent; however, it can be observed in cats with or without kidney diseases^53^. Other causes of renomegaly in cats include renal neoplasia (particularly lymphoma), perinephric pseudocysts, hydronephrosis, and polycystic kidney disease, which should be considered in the differential diagnosis^54^. The chi-square test results highlight significant changes in abdominal sonography findings during treatment for FIP with GS-441524, particularly regarding abdominal effusion, lymphadenopathy, and liver, spleen, and kidney alterations. These changes, except for the medullary rim sign in the kidneys, demonstrate a meaningful normalization process indicative of a positive response to treatment. While these findings are not exclusive to FIP, they have valid context for the disease’s resolution and progression during therapy. The new finding was the presence of crystals in the urinary bladder on the 56th and 83rd days of treatment in some cases. Allinder et al.^55^ reported urinary system calculi in two cats treated with GS-441524. The calculi in both cats demonstrated 98% similarity to GS‐441524. Substantial urine excretion of antivirals has been shown in humans, rodents, and dogs^56,57^. To our knowledge, crystal formation in cats undergoing antiviral treatment has not been reported; therefore, further investigation and careful attention to the urinary system should be considered during treatment with GS-441524 in FIP cats. The formation of urolithiasis or obstruction could impact the prognosis of the treatment. In our study, this condition was managed by changing to a urinary diet, adding a supplement, and sometimes administering fluid therapy and catheterization.

In the surveyed cats with thoracic radiographs, pleural effusion and mediastinal lymphadenopathy were the most frequently noted findings, aligning with effusive FIP pathophysiology. It is characterized by vasculitis, which causes protein-rich fluid to leak into body cavities, including the thoracic cavity, a defining disease feature^25^. Some findings, such as pneumonia, bronchitis, and cardiomegaly, may be considered incidental; however, further investigation should examine the virus’s impact on FIP cats’ cardiovascular and pulmonary systems. However, pyogranulomatous pneumonia and myocarditis can occur in FIP cats^14,25,58^. In this study, four cats with pneumonia and two cats with nodular patterns were reported in the initial phase of treatment, and all of them recovered by continuing the treatment process. All affected cats showed complete resolution of pleural effusion and regression of mediastinal lymphadenopathy by the end of treatment, highlighting the effectiveness of antiviral therapy in reversing these pathological changes and improving respiratory function and overall clinical recovery.

In this survey, both the starting and ending doses were higher for all disease forms compared to the previous study^4,7,28^. The average starting dosage for the wet form was 6.9 mg/kg, while the average ending dosage was 10.11 mg/kg. The mean dosages for starting and ending in the dry form were 9.65 mg/kg and 12.7 mg/kg, respectively. Ocular and neurologic forms were significantly more complicated to treat effectively and presented several management challenges for various reasons:

1. The blood–brain barrier in cats excludes about 80% of most drugs, while the blood-eye barrier excludes roughly 70%. These barriers also effectively block unwanted substances and agents, which can vary among individuals. Consequently, the extent of drug penetration is not entirely predictable^18^.
2. The efficiency of these barriers decreases in inflamed tissues and increases as inflammation subsides. This strategy is effective in the early stages of the disease but less beneficial for treatment in the later stages when only the virus remains after inflammation^18^.
3. The only way to increase drug levels in the brain or eyes is to elevate their concentrations in blood plasma by administering a higher dosage, either orally or parenterally, as there is no simple, safe, or effective method to reduce these barriers^18^.

These results emphasize the importance of tailoring dosages based on the specific type and stage of FIP, particularly in neurologic and ocular cases, to overcome the difficulties presented by biological barriers.

In this research, of the 259 cats exhibiting neurological and/or ocular symptoms, 239 were successfully cured, while two experienced a relapse and required additional treatment. One of these cats died five months after the second treatment due to sepsis and internal parasites. Consequently, our findings indicate that GS-441524, with a sufficient dosage, is a viable option for treating surviving ocular and/or neurologic FIP cats. In conclusion, some cats in this study exhibited mild and persistent neurological and ocular symptoms that did not improve, even with increased dosages and a treatment duration that extended beyond the initial 84 days. They continued to show normal appetite, activity levels, body temperature, and laboratory values at the publication time. These observations indicate that damage to the CNS or ocular structures may not always be fully reversible, underscoring the importance of beginning treatment before any damage becomes irreversible. As Timmann^43^ suggested, the occurrence of seizures in FIP indicates extensive brain damage and is often considered a sign of poor prognosis. One important sight a bout clinical signs of FIP is that they change over time^28^. For instance, in this study, some cats showed effusive or non-effusive form without neurologic signs and, at the cross of treatment, showed neurological symptoms, and the dosage and other disease management were considered based on neurological form. This aligns with the fact that in most cats, FCoV also invades the brain even without visible neurologic signs^59^.

According to the protocol outlined in this survey, the previous dosage was increased (+ 2–3 mg/kg) for all cats during the last three weeks of treatment. The main reason for these dosage adjustments stems from the author’s experience and observational data; this change was driven by evidence that some cats, despite showing improvement by day 56, experienced a clinical decline toward the end of treatment when the dosage remained constant. Furthermore, data shared by veterinarians in FIP Warrior groups on Facebook indicated that many cats had the potential for a cure with a dosage increase. Additionally, the relapse of FIP diminished with the protocols recommended in this study for GS-441524. For cats that did not exhibit a positive response to the standard dosage at any stage of treatment or in any disease form, increasing the dosage by + 2–3 mg/kg proved to be an effective strategy. However, Pedersen 2023 notes that if there is justification for raising the dosage, it should be increased by + 2 to + 5 mg/kg per day, contingent on the extent of remaining abnormalities and maintained for at least 4 weeks. Any dosage increase should be based on a positive response; a lack of response indicates potential issues such as the dosage being too low, developing drug resistance, ineffectiveness of the GS brand, misdiagnosis of FIP in the cat, or the presence of other complicating diseases^18^.

As mentioned, regular monitoring of four key factors—weight gain, clinical findings, laboratory parameters, and imaging factors— was essential for evaluating treatment response. As body weight increases, the dosage must be adjusted proportionally to maintain adequate drug levels and support continued recovery. Weight gain is essential for a positive response to treatment, particularly in kittens^18,19^. Clinical findings such as regression-related fever, icterus, lethargy, and decreased neurologic or ocular signs should be considered to enhance the cat’s overall freshness, activity, and appetite. Regarding imaging findings, it is important to consider the regression of fluid accumulation in its wet form and the decrease in abdominal lymph node sizes. Based on the original treatment protocol established by Pedersen^4,18^ and other studies, the recommended treatment duration for FIP is 84 days (12 weeks)^19,60^. In this research, 17.32% of patients had a treatment duration longer than this, while 0.8% received treatment for less than 84 days. Those who received less than 84 days could not continue the treatment due to their owners’ financial constraints. All of these patients are currently alive and recovered. However, the standard treatment duration for FIP in this study was 12 weeks. This survey proposes sterilizing intact cats during the final three weeks of treatment. The onset of estrus is stressful for cats and can potentially hinder recovery from FIP. Additionally, sex hormones, particularly androgens, negatively impact the immune system, increasing the risk of viral proliferation and mutation^28,61^.

Through the statistical analysis, risk factors, including gender, age, FIP form, fever, icterus, anemia, and thrombocytopenia, have prognostic factors affecting survival rates in FIP cats treated with GS-441524. Male cats show lower survival rates than females. This aligns with previous research indicating that male cats may have a greater risk of severe FIP due to hormonal influences or genetic predispositions^28^. The research shows that age had an effect on survival rate; that way, younger cats (under 12 months) often demonstrated the highest survival rates, whilst older cats (over 6 years) commonly showed the lowest. This tendency suggests that younger cats may exhibit better responses due to a lower prevalence of underlying health conditions. Nevertheless, the main reason is not apparent, as more FIP cats belong to the group under 12 months of age. The type of FIP significantly affected survival rates (*p* < 0.05), showing distinct differences among the effusive, non-effusive, neurological, ocular, and mixed forms. Effusive (abdominal and thoracic) FIP cats showed relatively high survival rates, indicating that the treatment can effectively manage pleural and peritoneal effusions while leading to fewer complications during treatment, besides early diagnosis based on the presence and analysis of the fluid accumulation in these cavities. In contrast, neurological and mixed FIP types had the lowest survival rates, likely due to delays in diagnosis and disease progression along with its complications, the severity of neurological involvement, and the difficulties associated with penetrating GS-441524 to the blood–brain barrier^18^. These findings emphasize the critical need for early intervention and appropriate dosing for successful treatment, particularly in neurological cats.

Several clinical markers were strongly associated with survival outcomes, including: (1) Fever was a significant predictor of survival (*p* = 0.0038), reinforcing the idea that persistent fever is linked to disease severity and poor prognosis. It is in contrast with another study that suggested nonsurvivors were significantly less likely to be febrile^62^. (2) Icterus (*p* = 0.0029) was a significant negative prognostic factor, with cats exhibiting jaundice having a higher likelihood of mortality. This issue has been presented by Katayama et al.^42^ that cats with bilirubin levels below 0.5 mg/dL had significantly better survival outcomes compared to those with levels above 4.0 mg/dL. In another study, bilirubin concentration (*P* = 0.001) was significantly increased in nonsurvivor cases^62^. Hyperbilirubinemia suggests that hemolysis is secondary to immune-mediated hemolytic anemia (IMHA); however, it must be severe to cause icterus. Bilirubin is sometimes elevated in cats with FIP without evidence of hemolysis, liver disease, or cholestasis. It has been speculated that the bilirubin metabolism and excretion into the biliary system are compromised in cats with FIP, similar to findings in sepsis^27,40^. This may contribute to worse outcomes, necessitating closer monitoring and supportive care. (3) Anemia (*p* = 0.0142) and thrombocytopenia (*p* < 0.05) were significantly associated with decreased survival, suggesting that hematological abnormalities are crucial in disease progression. In up to 65% of cats with FIP, anemia is present, typically with only a mild decrease in hematocrit. Anemia is mainly caused by secondary IMHA, in which autoantibodies against erythrocytes are produced, leading to hemolysis. This is another reason for anemia associated with chronic inflammation in FIP^63^. Tsai et al.^39^ established that the packed cell volume and bilirubin level were two predictors of disease staging and survival time. Thrombocytopenia is commonly observed in cats with FIP due to disseminated intravascular coagulation (DIC). Other parameters indicative of DIC, including fibrinogen degradation products (FDPs) and D-dimers, are also frequently elevated^27^. Approximately 50% of cats with FIP exhibit nonspecific reactive changes in the bone marrow upon necropsy^64^. These results highlight the critical role of early detection and proactive management of hematological complications in cats with FIP, potentially reducing these issues and improving treatment approaches for the disease as they are considered key indicators of survival factors.

Interestingly, certain variables initially suspected of influencing survival did not demonstrate a statistically significant impact: (1) Breed (*p* = 0.54): While different breeds showed varying survival rates, the differences were not statistically significant, indicating that breed alone does not strongly affect FIP. (2) Household type (*p* = 0.22): The type of household, whether single or multi-cat, did not influence survival, contradicting the hypothesis that multi-cat environments could contribute to disease severity. It just increases the risk of virus mutation and contamination (3) Living conditions (indoor vs. outdoor, *p* = 0.31): Survival rates were similar regardless of whether cats were kept indoors, outdoors, or had mixed access. It may be because all cats under treatment were indoors and had no outdoor access. (4) Concurrent diseases (*p* = 0.068): The presence of additional diseases with FIP cats (such as FeLV, FIV, or chronic inflammatory conditions) did not significantly influence survival rates. However, the response to treatment was more complex, as most cats in this survey undergoing extended treatment had coexisting diseases.

In the initial treatment phase, the injectable form was favored because many FIP cats experience gastrointestinal issues and complications related to their condition, including inappetence, vomiting, diarrhea, depression, and weakness, which can hinder oral drug absorption. Evidence from clinical findings indicates that cats in severe conditions gain better outcomes from parenteral administration, ensuring adequate bioavailability during the critical early stages of treatment. Following a specific treatment duration that differs for each cat based on their individual response, the therapy changes to the oral version of GS-441524. In this research, 21% (132 cats) maintained their treatment using tablets or capsules of GS-441524. The primary compliance issues associated with the injectable form of medication include pain, the risk of ulcers, and the stress that injection can cause for both cats and their owners. In contrast, oral treatment has several advantages, including being administered at home, eliminating the pain and stress of injections, and the need for daily moving to a veterinary clinic. Additionally, administering more than 2 cc via injection can be challenging and uncomfortable for the cat when dosages are increased. This makes the oral form a more favorable option. Despite its advantages, oral administration has drawbacks, such as concern with enteric absorption of the medication, the challenge of administering multiple capsules or tablets at the same time, and the risk of vomiting in cats. To decrease these concerns, it’s advised to fast cats for one hour before and after giving the medication, as noted by Anna-M Zuzzi-Krebitz in 2024^14^. Regarding adjunctive treatment in FIP, the infection tends to deplete the liver’s B12 stores through its inflammatory pathways. Additionally, vitamin B12 deficiency can lead to anemia, hypoxia, and malabsorption of nutrients^65^. Gabapentin can help the injection process by reducing stress and discomfort during injections^19,65^. The administration of corticosteroids should only be considered if there is an ongoing immune-mediated disease, such as immune-mediated hemolytic anemia (IMHA), which should be tapered and discontinued as soon as possible. In ocular form, ophthalmic corticosteroids can be utilized. Furthermore, it is essential to note that corticosteroids may offer supportive relief at best and are not curative^65^. In this study, the hepatic supplement was contemplated for the entire duration of the treatment period because of the elevated hepatic enzymes at initiation and throughout treatment, as well as the change in ultrasonographic findings, despite Dr.Pedersen suggesting that no additional treatments are necessary in cats with pure FIP disease^19^. Other adjuvant therapies, including fluid and antibiotic therapy, were considered based on the patient’s condition to manage the coexisting diseases (FPV, Mycoplasma hemophilis, etc.).

This study provides several noteworthy findings regarding the treatment of FIP with GS-441524:

1. This study presents results from GS-441524 treatments conducted on the largest population of cats with FIP infection to date. The large sample size enhances the reliability of the results, providing robust data on survival rates, relapse rates, and treatment efficacy across different forms of FIP.
2. *High survival rate and low overall mortality rate*: This study observed a survival rate of 94.12% (n = 592), representing one of the highest rates compared to prior studies reported by Pedersen^4^, Dickinson^8^, and Krentz^13^. Yin et al.^28^ and Jones et al.^17^ reported that 95.8% and 96.7% of cats treated with GS-441524 survived. However, their study populations were smaller compared to this study. The overall mortality rate is 6.48%, which is the lowest mortality rate in comparison with previous findings reports^4,9,12,42,60^.
3. *Low relapse rat*e: Only four cats (0.63%) experienced FIP relapse after treatment, which is fewer than reported in similar studies: 20%, 25.8%, 2.7%, 3.3%, and 10.8% in Morphy et al.^7^, Pedersen et al.^4^, Jones et al.^17^, and Yin et al.^28^.
4. *Long-term treatment success*: At the time of publication, 98.6% (n = 584) of the survived cats were still alive, which is significant and also indicates the sustained efficacy of GS-441524 therapy. This finding underscores the importance of long-term monitoring and post-treatment care to ensure continued remission and prevent late-onset complications.
5. *Impact on veterinary and owner decision*: The significantly high survival and low relapse rates observed in this study may encourage a greater willingness to pursue treatment, thereby reducing the number of cats euthanized or lost due to FIP. This addresses the most important concerns of the owners and veterinarians, as the cost of treatment is high and the treatment period is long.
6. *Efficacy in neurological and ocular FIP*: In addition to the reported percentages, more than 40% of the cats in the study experienced neuro- or ocular symptoms, which, along with the results mentioned above, indicates that the treatment protocol demonstrated a reasonable therapeutic response for severe and advanced FIP manifestations.
7. *Addressing key questions in FIP treatment*: Try to answer all of the questions regarding treating FIP with GS-441524 and provide critical insights into the different factors that could be important in diagnosing and surviving FIP cats.
8. In this study, the review of FIP cats conducted over four years encompassed a larger population and an extended follow-up period after treatment. Consequently, the findings from this study could enhance confidence in the treatment protocol, its efficacy, and safety concerning GS-441524, and the hope for a permanent cure. Additionally, it would be beneficial to conduct further investigations into the populations that were cured to evaluate the long-term effects of the treatment.

One of the limitations of this study was the absence of a definitive diagnosis to confirm dry FIP, and no necropsies were reported on deceased cats. Notably, despite lacking a definitive and least invasive diagnostic method, the response to treatment with GS-441524 could indicate FIP^59^. Many dry forms were identified in this study through this method. Another limitation was that many owners used multiple brands; this study did not determine whether all brands were effective or the actual amount of the active ingredient, GS-441524. As this drug is available on the black market, access to and cost of treatment pose significant challenges for pet owners. These factors are among the primary reasons some owners regret starting treatment, struggle to complete it, or neglect periodic examinations and paraclinical tests. Every effort was made to ensure that the treatment followed the protocol and was not terminated before final examinations and paraclinical tests were completed. This strategy sought to reduce the relapse rate significantly and increase the survival rate in comparison to another study. Nevertheless, as noted earlier, a small percentage could not complete the program due to financial constraints, owner neglect, or limited access to veterinary facilities at that time. Cytology was not conducted for every cat with abdominal lymphadenomegaly, which may have offered additional insights into the ongoing presence of enlarged abdominal lymph nodes. One of the main limitations of this study was the challenge of collecting comprehensive clinical, laboratory, and imaging data from a large study population over a four-year period. Therefore, I could not mention which devices were used to collect these data. In future studies, multivariable regression must still be employed to differentiate the impact of factors on treatment outcomes. Finally, although it is a retrospective study, it prefers a control or comparison group (e.g., untreated FIP cats or cats treated with supportive care), but I did not have it.

## Conclusion

This study is the initial investigation of GS-441524 FIP in Iran. Our results confirm that GS-441524 is a highly effective treatment for all forms of FIP, including effusive, non-effusive, neurological, and ocular forms, despite the historical challenges associated with managing FIP. Therapeutic success was observed in both oral and injectable administration. The treatment protocol used in this study resulted in a high cure rate (94.12%) and a low relapse rate (0.63%), further supporting the efficacy of GS-441524. Notably, this study involved the largest reported population of FIP-affected cats treated with GS-441524 to date, offering valuable insights into disease progression, laboratory and imaging changes during treatment, and factors influencing therapeutic outcomes, as well as several significant prognostic factors for survival in FIP cats. These findings add to the increasing evidence that supports GS-441524 as a dependable antiviral therapy for FIP. They are essential for approaching the foundation for future research to improve early diagnostic markers and evaluate long-term outcomes after treatment.

## Data Availability

The datasets used and/or analyzed during the current study are available from the corresponding author on reasonable request.
